# Efficacy of Chinese herbal medicine on nasal itching in children with allergic rhinitis: a systematic review and meta-analysis

**DOI:** 10.3389/fphar.2023.1240917

**Published:** 2023-08-23

**Authors:** Yuhang Chen, Jie Wang, Liqun Wu, Ye Zhang, Hong Chen, Ziwei Zhang

**Affiliations:** ^1^ The Second Clinical Medical College of Beijing University of Chinese Medicine, Dongfang Hospital of Beijing University of Chinese Medicine, Beijing, China; ^2^ The First Affiliated Hospital of Guizhou University of Traditional Chinese Medicine, Guiyang, Guizhou, China; ^3^ Dongfang Hospital of Beijing University of Chinese Medicine, Beijing, China

**Keywords:** Chinese herbal medicine (CHM), nasal itching, allergic rhinitis (AR), children, systematic review, meta-analysis

## Abstract

**Background:** Allergic rhinitis is prevalent among children and can cause nasal itching, fatigue, and even hinder growth and development. The main discomfort symptom of allergic rhinitis is nasal itching. Clinical reports suggest that Chinese herbal medicine (CHM) is effective in allergy rhinitis treatment. Therefore, we evaluate the clinical efficacy of Chinese herbal medicine in treating nasal itching caused by allergic rhinitis in children.

**Methods:** Nine databases, including PubMed, Embase, The Cochrane Library, Web of Science, China National Knowledge Infrastructure, Wan Fang Data, CQVIP, Chinese Biological Medicine, and ClinicalTrials.gov, were systematically searched from their inception until March 2023. Randomized controlled trials (RCTs) comparing the efficacy of Chinese herbal medicine, either alone or in combination with Western medicine, to Western medicine treatment or placebo intervention for treating allergic rhinitis in children were eligible for inclusion. The effectiveness of Chinese herbal medicines for nasal itching was mainly evaluated. The Risk of Bias tool 2.0 assessed the risk of bias. Statistical analysis using RevMan 5.3 and Stata/SE 12. The quality of evidence was evaluated by GRADEpro 3.6. Risk ratios (RR) with corresponding 95% confidence intervals (CI) were utilized to evaluate and present dichotomous data, while mean difference (MD) and standardized mean difference (SMD) were employed for continuous data. A fixed-effects model was applied in cases where the data exhibited homogeneity (*p* > 0.1, I2 < 50%), whereas a random-effects model was utilized for heterogeneous data. Statistical significance was determined by a *p*-value <0.05. This study was conducted by the Preferred Reporting Items for Systematic Reviews and Meta-Analyses (PRISMA) guidelines, and its review protocol was registered on the International Platform for Registered Systematic Reviews and Meta-Analysis Programs (INPLASY202340076).

**Results:** The review incorporated 23 studies. The meta-analysis indicated that herbal medicine was significantly related to the reduction of nasal itching (MD = −0.59, 95%CI: −0.94–0.24) and the increase of interleukin 10 level (SMD = 1.47, 95% CI: 0.90–2.05). Compared to Western medicine, the combining herbs and Western medicine showed better efficacy in relieving nasal itching, inhibiting immunoglobulin E, interleukin 4 and 33, enhancing interleukin 10, improving therapeutic efficiency, and reducing recurrent. Oral herbal medicine was more effective in treating nasal itching (MD = −0.45, 95% CI: −0.62–0.29). Combining oral and external herbal medicines was more efficient in treating nasal itching (MD = −0.44, 95% CI: −0.54–0.33), inhibiting immunoglobulin E, interleukin 4 (SMD = −0.87, 95% CI: −1.24–0.50) and 33 (SMD = −1.16, 95% CI: −1.54–0.77), and improving therapeutic efficiency. External herbal medicine did not show differences compared to Western medicines. Regarding safety, herbal medicine alone exhibited fewer adverse events than Western medicine; combining herbal and Western medicine showed no significant variation in adverse event incidence.

**Conclusion:** Chinese herbal medicine (CHM) holds great potential in alleviating symptoms, modulating immune factors levels, and reducing relapse in pediatric rhinitis. Meanwhile, CHM is relatively safe. However, the efficacy and safety of CHM in treating pediatric rhinitis still need to be confirmed due to the inclusion of studies with low methodological quality, small sample sizes, and potential heterogeneity. More high-quality research is necessary to provide reliable evidence for the clinical application of CHM.

**Systematic Review Registration**: INPLASY.com, identifier INPLASY202340076

## 1 Introduction

Allergic rhinitis is a prevalent condition in children, mediated by immunoglobulin E. Its prevalence is approximately 40% and gradually rising (Zhang and Zhang, 2019; Hox et al., 2020). In China, the current prevalence of allergic rhinitis among children is about 18.61% (Ruikun et al., 2022). The main symptoms include nasal itching, congestion, runny nose, and sneezing. Since there is a correlation between allergic diseases, allergic rhinitis is closely linked to other conditions such as asthma, upper airway cough syndrome, and cough variant asthma (Marple, 2010; Zvezdin et al., 2015; Donaldson, 2023). Additionally, allergic rhinitis can affect children’s nervous system, including attention deficit hyperactivity disorder and Tourette syndrome (Zhou et al., 2017; Xu et al., 2020; Liu X. et al., 2021). As a result, allergic rhinitis has become one of the research priorities in pediatric studies.

The pharmacological treatment of allergic rhinitis involves glucocorticoids, leukotriene receptor antagonists, antihistamines, and immunotherapy (Cobanoğlu et al., 2013; Klimek et al., 2019). These medications can have side effects such as impaired height growth, rhinorrhea, mental arousal, and drowsiness (Wolthers and Pedersen, 1993; Sastre et al., 2012; Mener et al., 2015; Marques et al., 2022), interfering with the standardized treatment of allergic rhinitis in children. Since allergic rhinitis can impede physical and intellectual development in children (He et al., 2017; Morais-Almeida et al., 2019; Sirufo et al., 2020), better management of its symptoms is required for optimal growth and development. Therefore, exploring alternative pharmacological therapies is necessary.

Chinese herbal medicine is a cornerstone of complementary alternative medicine, used in China for thousands of years. Herbal medicine can relieve allergic rhinitis nasal symptoms (Chan and Ng, 2020) by regulating inflammatory factors and immune function in affected children (Liu et al., 2022; Dou et al., 2023). Although some studies show the therapeutic effectiveness of herbal treatment in children with allergic rhinitis, meta-analyses examining the control of nasal symptoms from herbal medicine on allergic rhinitis in children are inconclusive. This review and meta-analysis aim to gather the appropriate evidence to comprehensively assess the overall therapeutic efficacy of herbal medicine on allergic rhinitis in children.

## 2 Materials and methods

This study adheres to the Preferred Reporting Items for Systematic Evaluations and Meta-Analyses (PRISMA) guidelines, and its synthesis protocol is registered on International Platform of Registered Systematic Review and Meta-analysis Protocols (INPLASY202340076).

### 2.1 Search strategies

Nine databases were searched from their creation to March 2023 included PubMed, Embase, The Cochrane Library, Web of Science, China National Knowledge Infrastructure, Wan Fang Data, CQVIP, Chinese Biological Medicine, and ClinicalTrials.gov. No language or country restrictions were applied. Medical subject terms combined with free terms enhanced our search parameters. Primary search terms were “herbal medicine,” “traditional Chinese medicine,” “allergic rhinitis,” “pediatric,” and “randomized.” Table 1 displays the search strategy utilized in PubMed.

**TABLE 1:** The search strategy of PubMed.

#1	“Drugs, Chinese Herbal” [Mesh]
#2	(((((Chinese Drugs, Plant [Title/Abstract]) OR (Chinese Herbal Drugs [Title/Abstract])) OR (Herbal Drugs, Chinese [Title/Abstract])) OR (Plant Extracts, Chinese [Title/Abstract])) OR (Chinese Plant Extracts [Title/Abstract])) OR (Extracts, Chinese Plant [Title/Abstract])
#3	1# or 2#
#4	“Rhinitis, Allergic” [Mesh]
#5	((Allergic Rhinitides [Title/Abstract]) OR (Rhinitides, Allergic [Title/Abstract])) OR (Allergic Rhinitis [Title/Abstract])
#6	4 or 5
#7	“Randomized Controlled Trials as Topic” [Mesh]
#8	((Clinical Trials, Randomized [Title/Abstract]) OR (Trials, Randomized Clinical [Title/Abstract])) OR (Controlled Clinical Trials, Randomized [Title/Abstract])
#9	7 or 8
#10	“Child” [Mesh]
#11	(“Child” [Mesh]) OR (Children [Title/Abstract])
#12	3# and 6# and 9# and 11#

### 2.2 Inclusion and exclusion criteria

Inclusion criteria: 1) children with allergic rhinitis diagnosed using clear diagnostic criteria (Nasal Group and Pediatrics Group, 2022), between the ages of 3–18 years; 2) randomized controlled trials; 3) compared Chinese herbal medicine (Including alone or in combination with western medicine) to Western medicine or placebo. No restrictions on the type, use, or duration of Chinese herbal medicine; 4) nasal itching score was reported in study.

Exclusion criteria were: 1) use acupuncture, massage, or any non-Chinese herbal treatments or control group treatment with Chinese medicine; 2) Children with other co-morbidities. Two independent reviewers (CYH and WJ) screened the studies based on the selection criteria. Any discrepancies between the assessments of these reviewers were resolved by a third reviewer (WLQ).

### 2.3 Types of outcome measures

The primary outcomes were nasal itching score (Gu and Dong, 2005), scored from 0–3 (The scoring system for itchy nose symptoms is as follows: a score of 0 indicates an absence of itchy nose symptoms, a score of 1 signifies occasional and intermittent itchy nose, a score of 2 represents a tolerable creeper sensation, and a score of 3 indicates the most severe level, characterized by an intolerable creeper sensation.). Secondary outcomes were efficiency, serum IgE levels, serum IL-4, IL-10, and IL-33 levels, recurrent rate, and adverse events.

### 2.4 Data extraction and bias assessment

Upon completing the literature search, we employed Endnote 20.0 software to manage the collected literature. Two reviewers (CYH and WJ) independently screened the identified studies’ titles, abstracts, and full texts using the predetermined inclusion and exclusion criteria. Essential information from the included studies, such as authors’ names, publication year, sample size, participant demographics (gender and age), intervention methods, outcome measures, and adverse effects, was extracted by the same two reviewers (CYH and WJ) utilizing a pre-established data collection form. Subsequently, this information was cross-validated by another two reviewers (CH and ZZW). In cases requiring additional details, one reviewer (ZY) proactively contacted the authors of specific studies via phone or email. Any reviewer disagreements will be resolved through discussion with another reviewer (WLQ). The Risk of Bias in included literature was evaluated by two independent reviewers (CYH and WJ) using the Risk of Bias 2 tool, which assessed six specific areas: randomization process, deviations from intended interventions, missing data, outcome measurement, selection of the reported result, and overall bias. After the data was extracted, we transformed raw continuous variable data into post-treatment minus pre-treatment delta values following guidelines proposed by Cochrane (JPT et al., 2022).

### 2.5 Evidence synthesis and statistical analysis

The statistical analysis of this study was conducted using Review Manager 5.3 software and STATA/SE 12.0. The quality of evidence was evaluated using GRADE profiler 3.6. Effect sizes for dichotomous data were analyzed using risk ratio (RR) and their 95% confidence interval (CI). Mean difference (MD) effect sizes with 95% confidence intervals were used to analyze nasal itching data. Standardized mean difference (SMD) with 95% confidence interval was used for continuous variables represented in different units as reported in the original studies. A fixed effects model was utilized to analyze data with good homogeneity (*I* ^*2*^ < 50%, *p* > 0.1), while a random effects model was used for data with poor homogeneity. *p* < 0.05 were considered to be statistically significant. Heterogeneity sources were elucidated by subgroup analysis when appropriate. Begg’s analysis was employed for studies with literature sizes equal to or greater than ten to ascertain publication bias. The stability of the study’s findings was determined using sensitivity analysis.

## 3 Results

### 3.1 Literature search results

**FIGURE 1:**
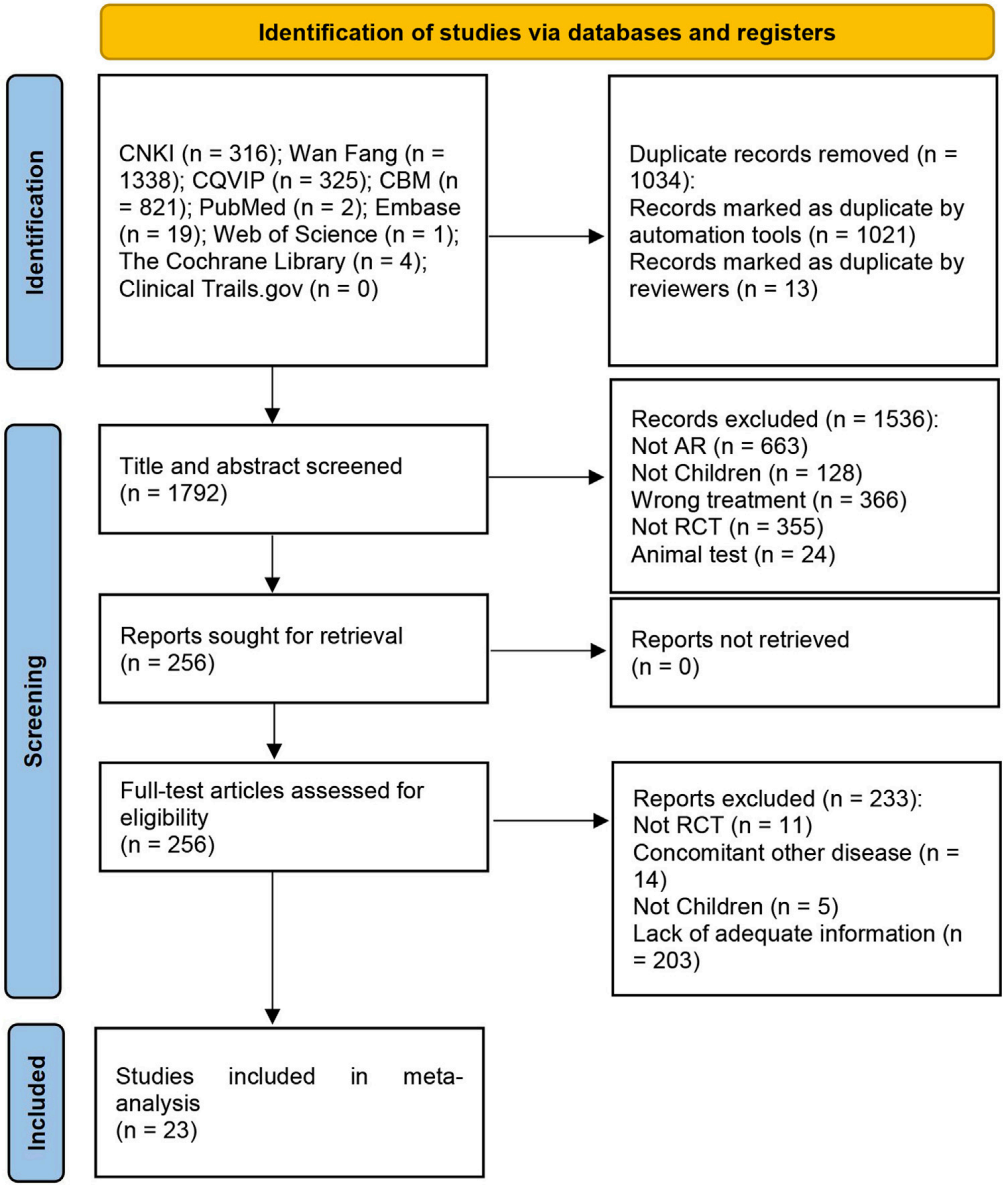
The process of study selection.

The initial search yielded 2,826 articles, from which 1,034 duplicates were identified and removed. A total of 1,536 articles were subsequently excluded based on their titles/abstracts according to the inclusion and exclusion criteria. After a full-text reading of the remaining 256 articles, 233 studies were excluded, leaving 23 RCTs (Huang et al., 2014; Wang and Zhao, 2016; Wang, 2017; Jiang et al., 2018; Chen, 2019; Lin, 2019; Shi et al., 2019; Wang et al., 2019; Yang et al., 2019; Yu and Wang, 2019; Zhang et al., 2019; Liu et al., 2020; Ma, 2020; Liu W. et al., 2021; Li and Guo, 2021; Sun et al., 2021; Zhang, 2021; Wang J. et al., 2022; Wang X. et al., 2022; Liu and Yang, 2022; Xu and Chen, 2022; Li et al., 2023; Wu et al., 2023) suitable for inclusion in this meta-analysis. Figure 1 illustrates the specific screening process, while Table 2 presents the distinctive characteristics of the analyzed studies. The drug utilization details, including dosage form, dose, and frequency, for each study and the duration of follow-up are presented in Table 2. Supplementary Table S1 provides additional information on the included studies’ patient sources, TCM syndromes, and funding sources. The characteristics of the included CHM are presented in Table 3.

**TABLE 2:** Characteristics of the included studies.

Study ID	Sample size		Course of disease		Mean age (year)		Male/Female (Male%)		Intervention		Course of treatment/Follow-up periods	Language	Outcomes
	Trial	Control	Trial	Control	Trial	Control	Trial	Control	Trial	Control			
Wu et al. (2023)	30	30	2.45 ± 0.858 y	2.50 ± 1.263 y	8.41 ± 1.843	8.55 ± 1.920	18/12	20/10	Biqiu Tongqiao Spray (one spray in each nostril qd) +Physiological Sea Water Nasal Spray (one spray in each nostril qd)	Mometasone Furoate Aqueous Nasal Spray (50 ug, one spray in each nostril qd) +Physiological Sea Water Nasal Spray (one spray in each nostril qd)	4 w/5 w	Chinese	①②
Huang et al. (2014)	66	66	NA	NA	9.36 ± 2.005	9.48 ± 2.032	32/34	34/32	Modified Buzhongyiqi Decoction (1 dose bid po)+Loratadine Tablets (5 mg qd po)+Montelukast Sodium Chewable Tablets (4 mg qn po)+Mometasone Furoate Aqueous Nasal Spray (50 ug, one spray in each nostril qd)	Loratadine Tablets (5 mg qd po)+Montelukast Sodium Chewable Tablets (4 mg qd po)+Mometasone Furoate Aqueous Nasal Spray (50 ug, one spray in each nostril qd)	30 d/30 d	Chinese	①②
Liu and Yang (2022)	60	60	3.29 ± 1.81 y	3.27 ± 1.82 y	8.87 ± 2.48	8.94 ± 2.51	32/28	36/24	Modified Bufei Decoction (100 mL bid po)+Cetirizine Dihydrochloride Oral Drops (5 mg–10 mg qd po)	Cetirizine Dihydrochloride Oral Drops (5 mg–10 mg qd po)	4w/4∼7 m	Chinese	①②③④⑦
Li et al. (2023)	52	52	2.91 ± 0.40 y	2.82 ± 0.37 y	7.51 ± 0.93	7.39 ± 0.81	34/18	37/15	Shenqi Xinyi Granule (0.5–1 sachet bid po)+Loratadine Sugar Syrup (5 mg–10 mg 30 kg qd po)	Loratadine Sugar Syrup (5 mg–10 mg 30kg qd po)	4w/4w	Chinese	①②③
Jiang et al. (2018)	70	70	7.03 ± 3.14 m	6.26 ± 2.72 m	6.69 ± 2.77	6.08 ± 2.39	34/36	37/33	Cangerzi Biyan Droppong Pill (14 pills tid po)+Montelukast Sodium Chewable Tablets (4 mg qd po)	Montelukast Sodium Chewable Tablets (4 mg qd po)	12w/12w	Chinese	①②③⑤
Wang J. et al. (2022)	30	30	NA	NA	4.97 ± 1.71	5.73 ± 2.35	22/8	19/11	Modified Guomin Decoction (100–200 mL bid po)	Montelukast Sodium Chewable Tablets (4 mg/10 mg qn po)	4w/4w	Chinese	①②⑥⑧
Shi et al. (2019)	30	30	NA	NA	NA	NA	NA	NA	Yupingfeng Granules (1 sachet bid po)+Dermatophagiodes Farinae Drops (1 drop qd po)	Dermatophagiodes Farinae Drops (1 drop qd po)	30days/6 m	Chinese	①②
Zhang (2021)	60	60	2.86 ± 0.94 y	2.87 ± 0.91 y	9.29 ± 3.62	9.25 ± 3.61	38/22	36/24	Shenling Baizhu Granule (1 sachet bid po)	Cetirizine Hydrochloride Syrup (2.5 mL–10 mL qd po)	15days/3 m	Chinese	①②
Yang et al. (2019)	33	36	8.12 ± 2.80 w	7.95 ± 2.70 w	8.52 ± 2.60	8.81 ± 2.72	19/14	20/16	Tongqiao Biyan Granule (0.5–1 sachet bid po)+Triamcinolone Acetonide Nasal Spray (1–2 spray in each nostril qd)	Triamcinolone Acetonide Nasal Spray (1–2 spray in each nostril qd)	4w/4w	Chinese	①②⑤⑧
Chen (2019)	53	53	3.23 ± 2.02 y	3.29 ± 2.08 y	9.02 ± 3.28	9.18 ± 3.63	30/23	28/25	Yiqi Tuomin Decoction (1 dose bid po)+Loratadine Tablets (4.4 mg–8.8 mg qd po)	Loratadine Tablets (4.4 mg–8.8 mg qd po)	3w/3w	Chinese	①②③⑤⑧
Ma (2020)	120	120	3.17 ± 0.80 y	3.09 ± 0.85 y	8.20 ± 1.39	8.11 ± 1.45	73/47	70/50	Bikang Tablets (4 tablets tid po)+Budesonide Nasal Spray (1 spray in each nostril bid)	Budesonide Nasal Spray (1 spray in each nostril bid)	4w/4w	Chinese	①②④
Wang et al. (2019)	61	61	3.24 ± 1.93 y	3.15 ± 1.86 y	7.13 ± 2.96	7.42 ± 3.16	47/14	49/12	Yiqi Yangyin Decoction (60mL–150mL bid po)+Biyan Transdermal (once every 3 days us.ext)	Cetirizine Dihydrochloride Oral Drops (4 mg–10 mg qn po)	3w/3w	Chinese	①②③④⑥⑧
Liu W. et al. (2021)	34	33	3.78 ± 2.68 y	3.83 ± 2.55 y	7.24 ± 2.33	7.38 ± 2.34	19/15	18/15	Xingbi Gelatin (1 drop in each nostril bid)	Budesonide Nasal Spray (64 ug spray in each nostril bid)	8w/8w	Chinese	①②③
Liu et al. (2020)	30	30	NA	NA	6.27 ± 2.78	5.86 ± 3.01	22/8	18/12	Bimin Tablets (1 tablet tid po)	Placebo (1 tablet tid po)	7d/1 y	Chinese	①②⑤⑦
Sun et al. (2021)	52	49	NA	NA	8	7	24/28	25/24	Modified Jiegeng Decoction (5 mL–20 mL tid po)	Loratadine Tablets (5 mg–10 mg qn po)	3w/3w	Chinese	①②
Lin (2019)	55	55	14.17 ± 4.23w	14.38 ± 4.14w	8.49 ± 2.31	8.74 ± 2.06	35/20	38/17	Biyuan Tongqiao Granule (15 g/1 sachet tid po)+Mometasone Furoate Aqueous Nasal Spray (100 ug/2 spray in each nostril bid)	Mometasone Furoate Aqueous Nasal Spray (100 ug/2 spray in each nostril bid)	8w/8w	Chinese	①②③
Wang X. et al. (2022)	150	150	36.81 ± 8.27 m	36.76 ± 8.21 m	7.58 ± 1.84	7.62 ± 1.91	86/64	86/64	Jianpi Tongqiao Decoction (100 mL bid po)+Loratadine Sugar Syrup (5 mg–10 mg qd po)	Loratadine Sugar Syrup (5 mg–10 mg qd po)	4w/4w	Chinese	①②③⑧
Wang (2017)	40	40	1.03 ± 0.41 y	1.01 ± 0.39 y	6.24 ± 1.65	6.27 ± 1.69	21/19	22/18	Tongqiao Biyan Granule (2 g/1 sachet tid po)+Cetirizine Dihydrochloride Oral Drops (0.5 mL qd/0.25 mL bid po)	Cetirizine Dihydrochloride Oral Drops (0.5 mL qd/0.25 mL bid po)	2 w/2 w	Chinese	①②⑧
Zhang et al. (2019)	80	80	1.49 ± 0.21 y	1.56 ± 0.17 y	8.85 ± 0.28	8.89 ± 0.26	44/36	41/39	Jianpi Qingfei Decoction (100 mL–200 mL bid po)+Moxibustion (qd us.ext)	Levocetirizine Dihydrochloride Oral Drops (5 mg qd po)	4 w/3 m	Chinese	①②③
Li and Guo (2021)	40	40	3.0 ± 1.6 y	2.9 ± 1.4 y	7.6 ± 2.9	7.5 ± 3.0	22/18	24/16	mild moxibustion (qod us.ext)+loratadine tablets (5 mg–10 mg qn po)	loratadine tablets (5 mg–10 mg qn po)	2 w/2 w	English	①②
Wang and Zhao (2016)	60	60	NA	NA	8.62 ± 2.07	8.34 ± 1.43	38/22	35/25	Cangxin Suspension (20 mL inh qd)+Montelukast Sodium Tablets (10 mg qd po)+Loratadine Tablets (10mg qd po)+Mometasone Furoate Aqueous Nasal Spray (200 ug/4 spray)	Montelukast Sodium Tablets (10mg qd po)+Loratadine Tablets (10 mg qd po)+Mometasone Furoate Aqueous Nasal Spray (200 ug/4 spray)	4w/4w	Chinese	①②⑥⑧
Xu and Chen (2022)	50	50	15.07 ± 4.75 m	14.41 ± 5.05 m	7.35 ± 2.70	7.47 ± 2.64	25/25	23/27	Xiaoqinglong Cangerzi Decoction (100 mL bid po)+Montelukast Sodium Chewable Tablets (4 mg/5 mg qd po)+Mometasone Furoate Aqueous Nasal Spray (50 ug/1 spray in each nostril qd)	Montelukast Sodium Chewable Tablets (4 mg/5 mg qd po)+Mometasone Furoate Aqueous Nasal Spray (50 ug/1 spray in each nostril qd)	18 d/18 d	Chinese	①②⑤⑥⑧
Yu and Wang (2019)	32	32	16.61 ± 1.35 m	16.62 ± 1.56 m	8.17 ± 0.64	8.42 ± 0.69	18/14	20/12	Sanao Cangerzi Decoction (75 mL–100 mL tid po)+Dermatophagiodes Farinae Drops (3 drops qd po)	Dermatophagiodes Farinae Drops (3 drops qd po)	4 w/4 w	Chinese	①②③

①, Nasal itching score; ②, Effective rate; ③, IgE: ④, IL4: ⑤, IL10; ⑥, IL33; ⑦, Recurrent rate; ⑧, Adverse reactions.

**TABLE 3:** The characteristic of CHM of included studies.

Study	Prescription name	Ingredients of herb prescription	Medicinals and dosages	Preparations	Source
Wu et al. (2023)	Biqiu Tongqiao Spray	xinyi, cangerzi, xiangbaizhi, boheye, fangfeng, cangzhu, dannanxing, xiakucao	The dried flower buds of Magnolia denudata Desr. (xin yi); The dried ripe fruit with total bracts of *Xanthium strumarium* L. (cang er zi); The dried root of Angelica dahurica (Hoffm.) Benth. & Hook.f. ex Franch. & Sav. (xiang bai zhi); The dried leaves of Mentha canadensis L. (bo he ye); The died roots of Saposhnikovia divaricata (Turcz. ex Ledeb.) Schischk. (fang feng); The died rhizome of Atractylodes lancea (Thunb.) DC. (cang zhu); The processed product of the dried rhizome of Arisaema erubescens (Wall.) Schott (dan nan xing); The dried fruit ears of Prunella vulgaris L. (xia ku cao)	Spray	Prepared by Chaoxia Wu et al.
Huang et al. (2014)	Modified Buzhongyiqi Decoction	huangqi 15 g, dangshen 15 g, baizhu 10 g, chenpi 5 g, danggui 10 g, shengma 10 g, chaihu 10 g, hezi 10 g, xixin 3 g, mahuang 5 g, yizhiren 10 g, zhigancao 10 g	The died roots of Astragalus mongholicus Bunge (huang qi) 15 g; The dried roots of Codonopsis pilosula (Franch.) Nannf. (dang shen) 15 g; The dried rhizomes of Atractylodes macrocephala Koidz. (bai zhu) 10 g; The dried ripe peel of Citrus × aurantium L. (chen pi) 5 g; The dried root of Angelica sinensis (Oliv.) Diels (dang gui) 10 g; The dried rhizomes of Actaea cimicifuga L. (sheng ma) 10 g; The dried roots of Bupleurum chinense DC. (chai hu) 10g; The died ripe fruits of Terminalia chebula Retz. (he zi) 10 g; The dried roots and rhizomes of Asarum heterotropoides F.Schmidt (xi xin) 3 g; The dried grassy stems of Ephedra sinica Stapf (ma huang) 5 g; The dry ripe fruits of Alpinia oxyphylla Miq. (yi zhi ren) 10 g; The processed product of the dried root and rhizome of Glycyrrhiza glabra L. (zhi gan cao) 10 g	Decoction	Prepared by Donghui Huang et al
Liu and Yang (2022)	Modified Bufei Decoction	danshen 9 g, huangqi 12 g, maidong 12 g, renshen 12 g, banxia 9 g, wuweizi 6 g, sangbaipi 12 g, zhigancao 6 g	The dried root and rhizome of Salvia miltiorrhiza Bunge (dan shen) 9 g; The dried root of Astragalus mongholicus Bunge (huang qi) 12 g; The dried tuber of Ophiopogon japonicus (Thunb.) Ker Gawl. (mai dong) 12 g; The dried roots and rhizomes of Panax ginseng C.A.Mey. (ren shen) 12 g; The dried tuber of Pinellia ternata (Thunb.) Makino (banxia) 9 g; The dried ripe fruits of Schisandra chinensis (Turcz.) Baill. (wu wei zi) 6 g; The dried root bark of Morus alba L. (sang bai pi) 12 g; The processed product of the dried root and rhizome of Glycyrrhiza glabra L. (zhi gan cao) 6 g	Decoction	Prepared by Fang Liu et al
Li et al. (2023)	Shenqi Xinyi Granules	huangqi 30g, dangshen 15g, jingmi 50g, xinyi 10 g	The died roots of Astragalus mongholicus Bunge (huang qi) 30 g; The dried root of Salvia miltiorrhiza Bge (dan shen) 15 g; The ripe fruits of Oryza sativa L. (jing mi) 50 g; The dried flower buds of Magnolia denudata Desr. (xin yi) 10 g	Granule	Prepared by Haijiao Li et al
Jiang et al. (2018)	Cangerzi Biyan Dropping Pills	cangerzi	The dried ripe fruit with total bracts of *Xanthium strumarium* L. (cang er zi)	Dropping Pill	Prepared by Hua Jiang et al
Wang J. et al. (2022)	Modified Guomin Decoction	yinchaihu 5 g, fangfeng 5 g, wumei 5 g, baizhu 5 g, baizhi 5 g, chuanxiong 5 g, xinyi 3 g, fuling 6g, huangqi 10 g, gancao 5 g	The dried root of Stellaria dichotoma var. lanceolata Bunge (yin chai hu) 5 g, fangfeng 5 g; The dried fruit of Prunus mume (Siebold) Siebold & Zucc. (wu mei) 5 g; The dried rhizome of Atractylodes macrocephala Koidz. (bai zhu) 5g; The dried root of Angelica dahurica (Hoffm.) Benth. & Hook.f. ex Franch. & Sav. (bai zhi) 5 g; The dried rhizome of Conioselinum anthriscoides ‘Chuanxiong’ (chuan xiong) 5 g; The dried flower buds of Magnolia denudata Desr. (xin yi) 3 g; The dried sclerotium of Poria cocos (Schw.) Wolf (fu ling) 6g; The dried root of Astragalus mongholicus Bunge (huang qi) 10g; The dried root and rhizome of Glycyrrhiza glabra L. (gan cao) 5 g	Decoction	Prepared by Jiana Wang et al.
Shi et al. (2019)	Yupingfeng Granules	huangqi, baizhu, fangfeng	The dried root of Astragalus mongholicus Bunge (huang qi); The dried rhizome of Atractylodes macrocephala Koidz. (bai zhu); The dried root of Saposhnikovia divaricata (Turcz.)Schischk (fang feng)	Granule	Prepared by Jiankai Shi et al
Zhang (2021)	Shenling Baizhu Granules	baizhu 12 g, shanyao 12 g, taizishen 9 g, chenpi 12 g, baibiandou 10g, xinyi 12g, fangfeng 12 g, shengjiang 9 g, sharen 6 g, fuling 12 g, lianzi 10 g, jiegeng 9 g, yiyiren 12 g, guizhi 9 g, gancao 3 g	The dried rhizome of Atractylodes macrocephala Koidz. (bai zhu) 12 g; The dried rhizome of Dioscorea oppositifolia L. (shan yao) 12 g; The dried tuberous root of Pseudostellaria heterophylla (Miq.) Pax (tai zi shen) 9 g; The dried ripe peel of Citrus × aurantium L. (chen pi) 12 g; The dried ripe seed of Dolichos lablab L. (bai bian dou) 10 g; The dried flower buds of Magnolia denudata Desr. (xin yi) 12 g; The died roots of Saposhnikovia divaricata (Turcz. ex Ledeb.) Schischk. (fang feng) 12 g; The dried rhizome of Zingiber officinale Roscoe (sheng jiang) 9 g; The dried ripe fruit of Amomum villosum Lour. (sha ren) 6g; The dried sclerotium of Poria cocos (Schw.) Wolf (fu ling) 12 g; The dried ripe seed of *Nelumbo nucifera* Gaertn (lian zi) 10 g; The dried root of Platycodon grandiflorum (Jacq.)A.DC. (jie geng) 9g; The dried ripe kernel of Coix lacryma-jobi L. (yi yi ren) 12 g; The dried twigs of Neolitsea cassia (L.) Kosterm. (gui zhi) 9g; The dried root and rhizome of Glycyrrhiza glabra L. (gan cao) 3 g	Granule	Prepared by Junxi Zhang et al.
Yang et al. (2019)	Tongqiao Biyan Granules	xinyi, huangqi, baizhi, cangerzi, fangfeng	The dried flower buds of Magnolia denudata Desr. (xin yi); The dried root of Astragalus mongholicus Bunge (huang qi); The dried root of Angelica dahurica (Hoffm.) Benth. & Hook.f. ex Franch. & Sav. (bai zhi); The dried ripe fruit with total bracts of *Xanthium strumarium* L. (cang er zi); The died roots of Saposhnikovia divaricata (Turcz. ex Ledeb.) Schischk. (fang feng)	Granule	Chengdu Dikang Technology Pharmaceutical Stock Co., Ltd.
Chen (2019)	Yiqi Tuomin Decoction	huangqi 30 g, fangfeng 10 g, chantui 10 g, xixin 3 g	The dried root of Astragalus mongholicus Bunge (huang qi) 30 g; The died roots of Saposhnikovia divaricata (Turcz. ex Ledeb.) Schischk. (fang feng) 10 g; The exfoliated shells of Cicadae Periostracum (chantui) 10 g; The dried roots and rhizomes of Asarum heterotropoides F.Schmidt (xi xin) 3 g	Decoction	Prepared by Shuang Chen et al.
Ma (2020)	Bikang Teblets	xinyi, rendongteng, dahuang, bohe	The dried flower buds of Magnolia denudata Desr. (xin yi); The dried stems and branches of *Lonicera japonica* Thunb. (ren dong teng); The dried root and rhizome of Rheum palmatum L. (da huang); The dried stems and leaves of Mentha canadensis L. (bo he)	Teblets	Guizhou Guangzheng Pharmaceutical Co.,Ltd
Wang et al. (2019)	Yiqi Yangyin Decoction	huangqi 30 g, nanshashen 15 g, shanyao 12 g, digupi 10 g, huangjing 10 g, wumei 12 g, fangfeng 10 g, zhigancao 6 g; jiezi, xixin, yanhusuo, shengbanxia, gansui	The dried root of Astragalus mongholicus Bunge (huang qi) 30 g; The dried roots of Adenophora triphylla (Thunb.) A.DC. (nan sha shen) 15 g; The dried rhizome of Dioscorea oppositifolia L. (shan yao) 12 g; The dried root bark of Lycium barbarum L. (di gu pi) 10 g; The dried rhizome of Polygonatum sibiricum Redouté (huang jing) 10g; The dried fruit of Prunus mume (Siebold) Siebold & Zucc. (wu mei) 12 g; The died roots of Saposhnikovia divaricata (Turcz. ex Ledeb.) Schischk. (fang feng) 10g; The processed product of the dried root and rhizome of Glycyrrhiza glabra L. (zhi gan cao) 6g; The dried mature seeds of Brassica juncea (L.) Czern. (jie zi); The dried roots and rhizomes of Asarum heterotropoides F.Schmidt (xi xin); The dried tubers of Corydalis yanhusuo (Y.H.Chou & Chun C.Hsu) W.T.Wang ex Z.Y.Su and C.Y.Wu (yan hu suo); The dried tuber of Pinellia ternata (Thunb.) Makino (ban xia); The dried tubers of Euphorbia kansui S.L.Liou ex S.B.Ho (gan sui)	Decoction	Prepared by Wei Wang et al.
Liu W. et al. (2021)	Xingbi Gelatin	xuchangqin, chantui, niuhuang, bingpian	The ried rhizome of Cynanchum paniculatum (Bge.) Kitag. (xu chang qing); The exfoliated shells of Cicadae Periostracum (chan tui); The dried gall-stone of *Bos taurus* domesticus Gmelin (niu huang); The processed product of Cinnamomum cam phora L.) Presl (bing pian)	Gelatin	Prepared by Wen Liu et al
Liu et al. (2020)	Bimin Tablets	huangqi 10 g, fangfeng 10 g, baizhu 10 g, xinyi 10 g, cangerzi 10 g, paojiang 10 g, gancao 10 g	The dried root of Astragalus mongholicus Bunge (huang qi) 10 g; The died roots of Saposhnikovia divaricata (Turcz. ex Ledeb.) Schischk. (fang feng) 10 g; The dried rhizome of Atractylodes macrocephala Koidz. (bai zhu) 10 g; The dried flower buds of Magnolia denudata Desr. (xin yi) 10g; The dried ripe fruit with total bracts of *Xanthium strumarium* L. (cang er zi) 10 g; The processed product of of the dried rhizome of Zingiber officinale Roscoe (pao jiang) 10 g; The dried root and rhizome of Glycyrrhiza glabra L. (gan cao) 10 g	Tablets	Prepared by Xiang Liu et al.
Sun et al. (2021)	Modified Jiegeng Decoction	xuanshen 6 g, rendongteng 5 g, bohe 5 g, guanghuoxiang 5g, gancao 3 g, huangqin 3 g, zhuyechaihu 3 g, chantui 3 g, jiegeng 3 g, juhua 3 g, xinyi 3 g	The dried roots of Scrophularia ningpoensis Hemsl. (xuan shen) 6 g; The dried stems and branches of *Lonicera japonica* Thunb. (ren dong teng) 5 g; The dried stems and leaves of Mentha canadensis L. (bo he) 5 g; The dried aboveground part of Pogostemon cablin (Blanco) Benth (guang huo xiang) 5 g; The dried root and rhizome of Glycyrrhiza glabra L. (gan cao) 3 g; The dried roots of Scutellaria baicalensis Georgi (huang qin) 3 g; The dried roots of Bupleurum chinense DC. (zhu ye chai hu) 3 g; The exfoliated shells of Cicadae Periostracum (chan tui) 3g; The dried root of Platycodon grandiflorum (Jacq.)A.DC. (jie geng) 3g; The dried flower of Chrysanthemum × morifolium (Ramat.) Hemsl. (ju hua) 3 g; The dried flower buds of Magnolia denudata Desr. (xin yi) 3 g	Decoction	Prepared by Xiangjuan Sun et al.
Lin (2019)	Biyuan Tongqiao Granules	xinyi, mahuang, cangerzi, gaoben, bohe, baizhi, tianhuafen, yejuhua, lianqiao, huangqin, fuling, danshen, gancao	The dried flower buds of Magnolia denudata Desr. (xin yi); The dried herbaceous stems of Ephedra sinica Stapf (ma huang); The dried ripe fruit with total bracts of *Xanthium strumarium* L. (cang er zi); The dried rhizomes and roots of Conioselinum anthriscoides (H.Boissieu) Pimenov & Kljuykov (gao ben); The dried stems and leaves of Mentha canadensis L. (bo he); The dried root of Angelica dahurica (Hoffm.) Benth. and Hook.f. ex Franch. and Sav. (bai zhi); The dried roots of Trichosanthes kirilowii Maxim. (tian hua fen); The dried flower of Chrysanthemum indicum L. (ye ju hua); The dried fruits of Forsythia suspensa (Thunb.) Vahl (lian qiao); The dried roots of Scutellaria baicalensis Georgi (huang qin); The dried sclerotium of Poria cocos (Schw.) Wolf (fu ling); The dried root and rhizome of Salvia miltiorrhiza Bunge; The dried root and rhizome of Glycyrrhiza glabra L. (gan cao)	Granule	Shandong New Time Pharmaceutical Co., Ltd.
Wang X. et al. (2022)	Jianpi Tongqiao Decoction	huangqi 10 g, fangfeng 10 g, baishao 10 g, baizhu 10 g, guizhi 10 g, xinyi 10 g, chaihu 10 g, danggui 10 g, mahuang 6 g, baizhi 6g, cangerzi 6 g, wumei 6g, gancao 3 g	The dried root of Astragalus mongholicus Bunge (huang qi) 10 g; The died roots of Saposhnikovia divaricata (Turcz. ex Ledeb.) Schischk. (fang feng) 10 g; The dried root of Paeonia lactiflora Pall. (bai shao) 10 g. The dried rhizome of Atractylodes macrocephala Koidz. (bai zhu) 10g; The dried shoots of Neolitsea cassia L.) Kosterm. (gui zhi) 10g; The dried flower buds of Magnolia denudata Desr. (xin yi) 10g, chaihu 10g; The dried root of Angelica sinensis (Oliv.) Diels (dang gui) 10g; The dried herbaceous stems of Ephedra sinica Stapf (ma huang) 6 g; The dried root of Angelica dahurica (Hoffm.) Benth. and Hook.f. ex Franch. and Sav. (bai zhi) 6g; The dried ripe fruit with total bracts of *Xanthium strumarium* L. (cang er zi) 6 g; The dried fruit of Prunus mume (Siebold) Siebold & Zucc. (wu mei) 6 g; The dried root and rhizome of Glycyrrhiza glabra L. (gan cao) 3 g	Decoction	Prepared by Xiumin Wang et al.
Wang (2017)	Tongqiao Biyan Granules	xinyi, huangqi, baizhi, cangerzi, fangfeng, baizhu, bohe	The dried flower buds of Magnolia denudata Desr. (xin yi); The dried root of Astragalus mongholicus Bunge (huang qi); The dried root of Angelica dahurica (Hoffm.) Benth. & Hook.f. ex Franch. & Sav. (bai zhi); The dried ripe fruit with total bracts of *Xanthium strumarium* L. (cang er zi); The died roots of Saposhnikovia divaricata (Turcz. ex Ledeb.) Schischk. (fang feng); The dried rhizome of Atractylodes macrocephala Koidz. (bai zhu); The dried stems and leaves of Mentha canadensis L. (bo he)	Granule	Prepared by Yan Wang et al.
Zhang et al. (2019)	Jianpi Qingfei Decoction	xinyi 10 g, fangfeng 10 g, jiegeng 10 g, mahuang 1 g, chuanxiong 5 g, zhizi 5 g, xingren 5 g, gancao 3 g	The dried flower buds of Magnolia denudata Desr. (xin yi) 10 g; The died roots of Saposhnikovia divaricata (Turcz. ex Ledeb.) Schischk. (fang feng) 10 g; The dried root of Platycodon grandiflorum (Jacq.)A.DC. (jie geng) 10 g; The dried herbaceous stems of Ephedra sinica Stapf (ma huang) 1g; The dried rhizome of Conioselinum anthriscoides “Chuanxiong” (chuan xiong) 5 g; The dried ripe fruits of Gardenia jasminoides J.Ellis (zhi zi) 5g; The dried ripe seed of Prunus armeniaca L. (ku xing ren) 5g; The dried root and rhizome of Glycyrrhiza glabra L. (gan cao) 3 g	Decoction	Prepared by Ying Zhang et al
Li and Guo (2021)	Mild Moxibustion	aiye	The dried Dry leaves of Artemisia argyi H.Lév. & Vaniot (ai ye)	Moxibustion	Prepared by Yong Li et al.
Wang and Zhao (2016)	Cangxin Spray	cangerzi 10 g, xinyihua 5 g, baizhi 10 g, huangqin 10 g, beiqi 20 g, bohe 5 g, chuanxiong 5 g, zhebeimu 10 g, gancao 5 g, juhua 10 g, dandouchi 10 g	The dried ripe fruit with total bracts of *Xanthium strumarium* L. (cang er zi) 10g; The dried flower buds of Magnolia denudata Desr. (xin yi hua) 5g; The dried root of Angelica dahurica (Hoffm.) Benth. and Hook.f. ex Franch. & Sav. (bai zhi) 10 g; The dried roots of Scutellaria baicalensis Georgi (huang qin) 10 g; The dried root of Astragalus mongholicus Bunge (bei huang qi) 20 g; The dried stems and leaves of Mentha canadensis L. (bo he) 5 g; The dried rhizome of Conioselinum anthriscoides ‘Chuanxiong’ (chuan xiong) 5 g; The dried bulbs of Fritillaria thunbergii Miq. (zhe bei mu) 10 g; The dried root and rhizome of Glycyrrhiza glabra L. (gan cao) 5 g; The dried flower of Chrysanthemum × morifolium (Ramat.) Hemsl. (ju hua) 10 g; The dried ripe seeds of Glycine max (L.) Merr. (dan dou chi) 10 g	Spray	Prepared by Yuan Wang et al.
Xu and Chen (2022)	Xiaoqinglong Cangerzi Decoction	baizhi 9 g, shaoyao 9 g, xinyi 9 g, banxia 9 g, wuweizi 6 g, guizhi 6 g, cangerzi 6 g, zhigancao 6 g, mahuang 6 g, bohe 3 g, xinyi 3 g, ganjiang 3 g	The dried root of Angelica dahurica (Hoffm.) Benth. & Hook.f. ex Franch. & Sav. (bai zhi) 9 g; The dried root of Paeonia lactiflora Pall. (shao yao) 9 g; The dried flower buds of Magnolia denudata Desr. (xin yi) 9g; The dried processed tuber of Pinellia ternata (Thunb.) Makino (ban xia) 9 g; The dried ripe fruit of Schisandra chinensis (Turcz.) Baill. (wu wei zi) 6 g; The dried shoots of Neolitsea cassia (L.) Kosterm. (gui zhi) 6 g; The dried ripe fruit with total bracts of *Xanthium strumarium* L. (cang er zi) 6 g; The processed product of the dried root and rhizome of Glycyrrhiza glabra L. (zhi gan cao) 6 g; The dried herbaceous stems of Ephedra sinica Stapf (ma huang) 6 g; The dried stems and leaves of Mentha canadensis L. (bo he) 3 g; The dried roots and rhizomes of Asarum heterotropoides F.Schmidt (xi xin) 3 g; The dried rhizome of Zingiber officinale Roscoe (gan jiang) 3 g	Decoction	Prepared by Yuan Xu et al.
Yu and Wang (2019)	Sanao Cangerzi Decoction	bohe 6 g, chaocangerzi 10 g, mimahuang 6 g, baizhi 6 g, kuxingren 10 g, xinyi 6 g, gancao 3 g	The dried stems and leaves of Mentha canadensis L. (bo he) 6 g; The processed product of the dried ripe fruit with total bracts of *Xanthium strumarium* L. (chao cang er zi) 10 g; The processed product of the dried herbaceous stems of Ephedra sinica Stapf (mi ma huang) 6 g; The dried root of Angelica dahurica (Hoffm.) Benth. & Hook.f. ex Franch. & Sav. (bai zhi) 6 g; The dried ripe seed of Prunus armeniaca L. (ku xing ren) 10g; The dried flower buds of Magnolia denudata Desr. (xin yi) 6 g; The dried root and rhizome of Glycyrrhiza glabra L. (gan cao) 3 g	Decoction	Prepared by Zhou Yu et al

Nine of the 23 randomized controlled trials (RCTs) explicitly stated that the patients were sourced from the outpatient clinic. One RCT included patients from the outpatient clinic and inpatient wards, while the remaining 13 did not provide explicit information regarding the patient source (Supplementary Table S1). In the 23 RCTs, one RCT (Liu et al., 2020) compared oral Chinese herbal medicine with placebo. Seven RCTs compared Chinese herbal medicine *versus* Western medicine, including three (Sun et al., 2021; Zhang, 2021; Wang J. et al., 2022) studies using oral CHM, two (Liu W. et al., 2021; Wu et al., 2023) using external CHM, and two studies (Wang et al., 2019; Zhang et al., 2019) using a combination of oral and external CHM. Fifteen RCTs used CHM in combination with WM compared with WM, of which thirteen RCTs (Huang et al., 2014; Wang and Zhao, 2016; Wang, 2017; Jiang et al., 2018; Chen, 2019; Lin, 2019; Shi et al., 2019; Yang et al., 2019; Yu and Wang, 2019; Ma, 2020; Wang X. et al., 2022; Liu and Yang, 2022; Li et al., 2023) used oral CHM and two RCTs (Wang and Zhao, 2016; Li and Guo, 2021) used external CHM. All 23 RCTs reported nasal itching score and efficiency rate. With serum IgE levels mentioned in ten (Jiang et al., 2018; Chen, 2019; Lin, 2019; Wang et al., 2019; Yu and Wang, 2019; Zhang et al., 2019; Liu W. et al., 2021; Wang X. et al., 2022; Liu and Yang, 2022; Li et al., 2023), IL-4 in two (Wang et al., 2019; Liu and Yang, 2022), IL-10 in five (Jiang et al., 2018; Chen, 2019; Yang et al., 2019; Liu et al., 2020; Xu and Chen, 2022) and IL33 in two (Wang et al., 2019; Xu and Chen, 2022). Recurrent rates were noted in two RCTs (Liu et al., 2020; Liu and Yang, 2022), while adverse events were reported in eight RCTs (Wang and Zhao, 2016; Wang, 2017; Chen, 2019; Wang et al., 2019; Yang et al., 2019; Wang J. et al., 2022; Wang X. et al., 2022; Xu and Chen, 2022). Of the 23 RCTs, 12 RCTs (Chen, 2019; Shi et al., 2019; Wang et al., 2019; Zhang et al., 2019; Liu W. et al., 2021; Li and Guo, 2021; Sun et al., 2021; Zhang, 2021; Wang J. et al., 2022; Wang X. et al., 2022; Li et al., 2023; Wu et al., 2023) indicated TCM syndromes, of which 2 RCTs (Sun et al., 2021; Wu et al., 2023) had a TCM syndrome of Lung meridian latent heat syndrome, 1 RCT (Li et al., 2023) was Spleen qi deficiency syndrome, 3 RCTs (Chen, 2019; Li and Guo, 2021; Wang J. et al., 2022) had a Lung-qi deficiency cold pattern, 4 RCTs (Shi et al., 2019; Zhang et al., 2019; Zhang, 2021; Wang X. et al., 2022) were Pulmonosplenic asthenia, 1 RCT (Wang et al., 2019) was Deficiency of both vital energy and yin, and 1 RCT (Liu W. et al., 2021) was Syndrome of wind invading the lung (Supplementary Table S1).

### 3.2 Risk of bias assessment

**FIGURE 2:**
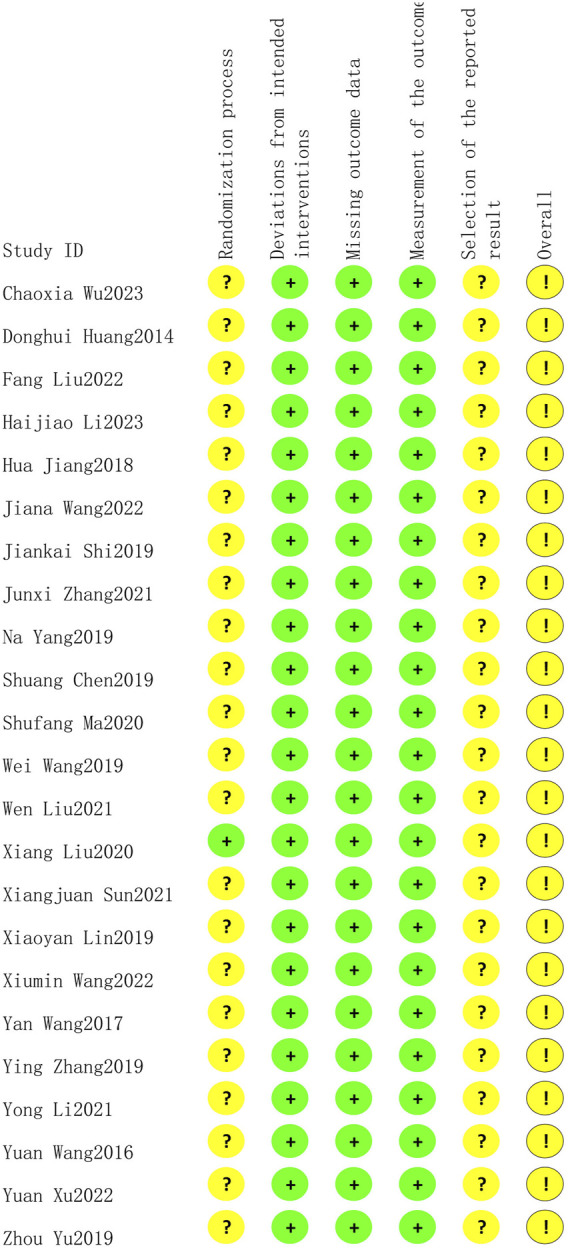
The result for the evaluation of selected studies by ROB2.0.

**FIGURE 3:**
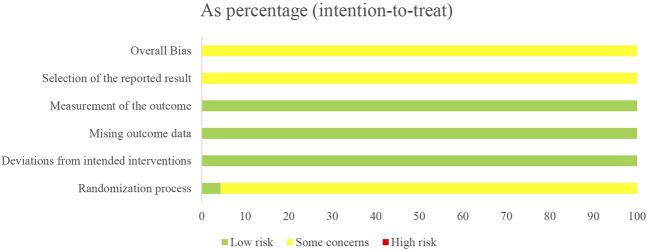
The result for the evaluation of selected studies by ROB2.0.

Only one study (Liu et al., 2020) reported low risk of randomization as the utilized of blinding, other studies did not utilize blinding so reported moderate risk of bias. All twenty-three studies lacked specification on study enrollment information, resulting in a moderate risk of selection of reported bias. All twenty-three studies reported low risk of deviations from intended intervention, missing outcome date, and measurement of outcome. The overall risk of bias for all twenty-three studies was moderate. The risk of article bias is presented in Figures 2, 3.

### 3.3 Primary outcomes

#### 3.3.1 Nasal itching

##### 3.3.1.1 Chinese herbal medicine *versus* placebo

**FIGURE 4:**
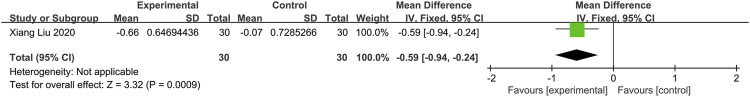
The result of meta-analysis of CHM *versus* placebo on nasal itching.

One study (Liu et al., 2020) compared herbal medicine to placebo. The fixed-effects model analysis showed that Chinese herbal medicine is significantly related to an alleviation of nasal itching (*n* = 60; MD = −0.59, 95%CI: −0.94 to −0.24, *p* = 0.0009; Figure 4).

##### 3.3.1.2 Chinese herbal medicine *versus* western medicine

###### 3.3.1.2.1 Oral CHM

**FIGURE 5:**
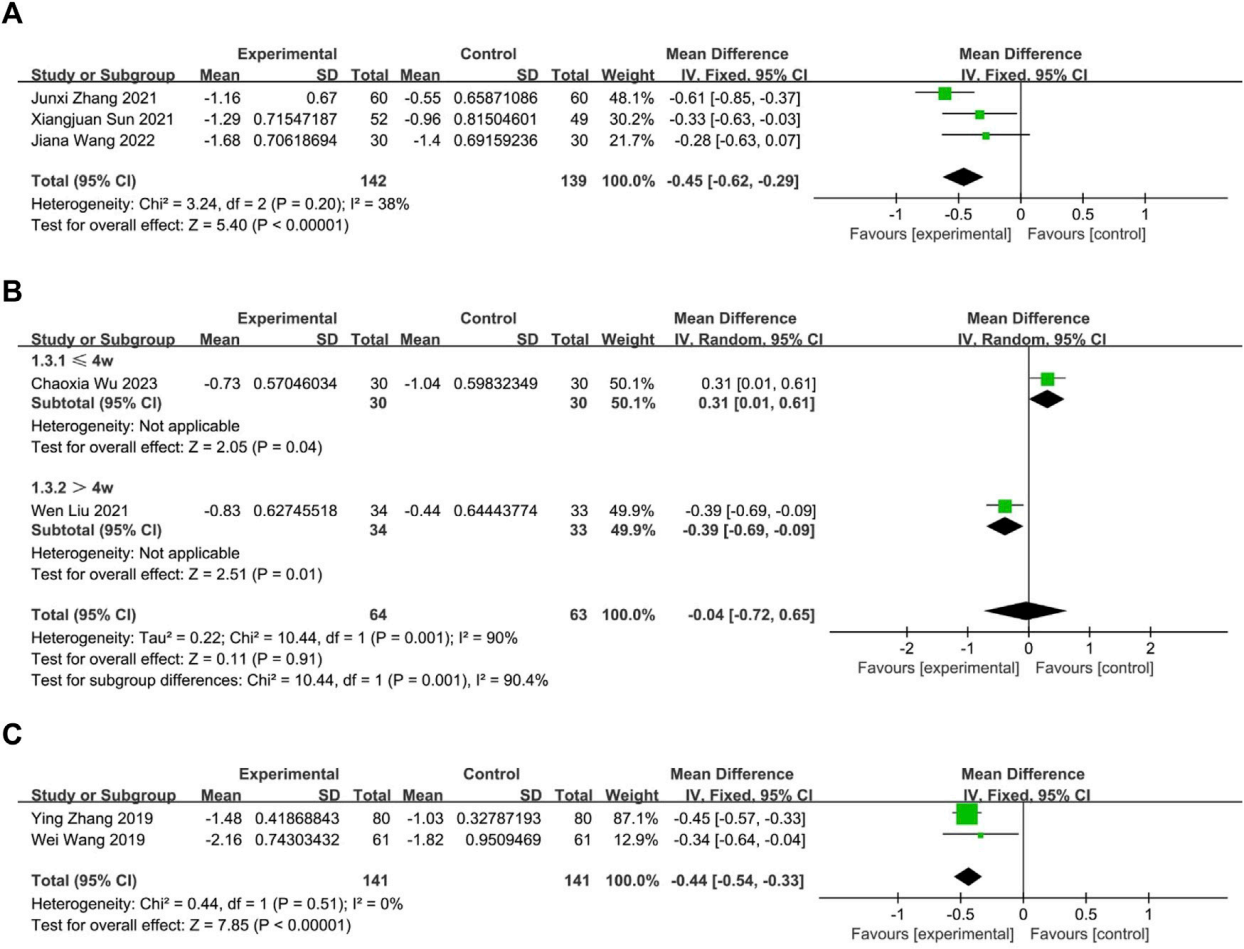
The result of meta-analysis of CHM *versus* WM on nasal itching. **(A)** oral CHM; **(B)** external CHM; **(C)** combination of oral and external CHM.

Three RCTs (Sun et al., 2021; Zhang, 2021; Wang J. et al., 2022) compared the effects of oral Chinese herbal medicine to Western medicine, all of which had no pharmacological intervention lasting longer than 4 weeks. Fixed-effects model (*p* = 0.20, I2 = 38%), CHM demonstrated a statistically significant advantage in the relief of nasal itching (*n* = 281, MD = −0.45, 95% CI: −0.62 to −0.29, *p* < 0.00001; Figure 5A).

###### 3.3.1.2.2 External CHM

Two RCTs (Liu W. et al., 2021; Wu et al., 2023) compared the effects of external Chinese herbal medicine to Western medicine. A random-effects model analysis (*p* = 0.001, I2 = 90%) revealed no significant difference between the two treatments (n = 127, MD = −0.04, 95% CI: −0.72 to 0.65, *p* = 0.91; Figure 5B). However, subgroup analysis based on intervention duration showed that one RCT with an intervention lasting more than 4 weeks favored external CHM over WM (n = 67, MD = −0.39, 95% CI: −0.69 to −0.09, *p* = 0.01; Figure 5B), while another RCT with an intervention lasting no more than 4 weeks reported better efficacy for WM than CHM (*n* = 60, MD = 0.31, 95% CI: 0.01 to 0.61, *p* = 0.04; Figure 5B).

###### 3.3.1.2.3 Oral and external CHM

Two trials (Wang et al., 2019; Zhang et al., 2019) compared the combination of oral and external CHM to Western medicine. None of the interventions lasted longer than 4 weeks. Based on a fixed-effects model analysis (*p* = 0.51, I2 = 0%), Chinese herbal medicine demonstrated a statistically significant advantage over WM in treating nasal itching (*n* = 282, MD = −0.44, 95% CI: −0.54 to −0.33, *p* < 0.00001; Figure 5C).

##### 3.3.1.3 Combination of CHM and WM *versus* WM alone

###### 3.3.1.3.1 Oral CHM treatment

**FIGURE 6:**
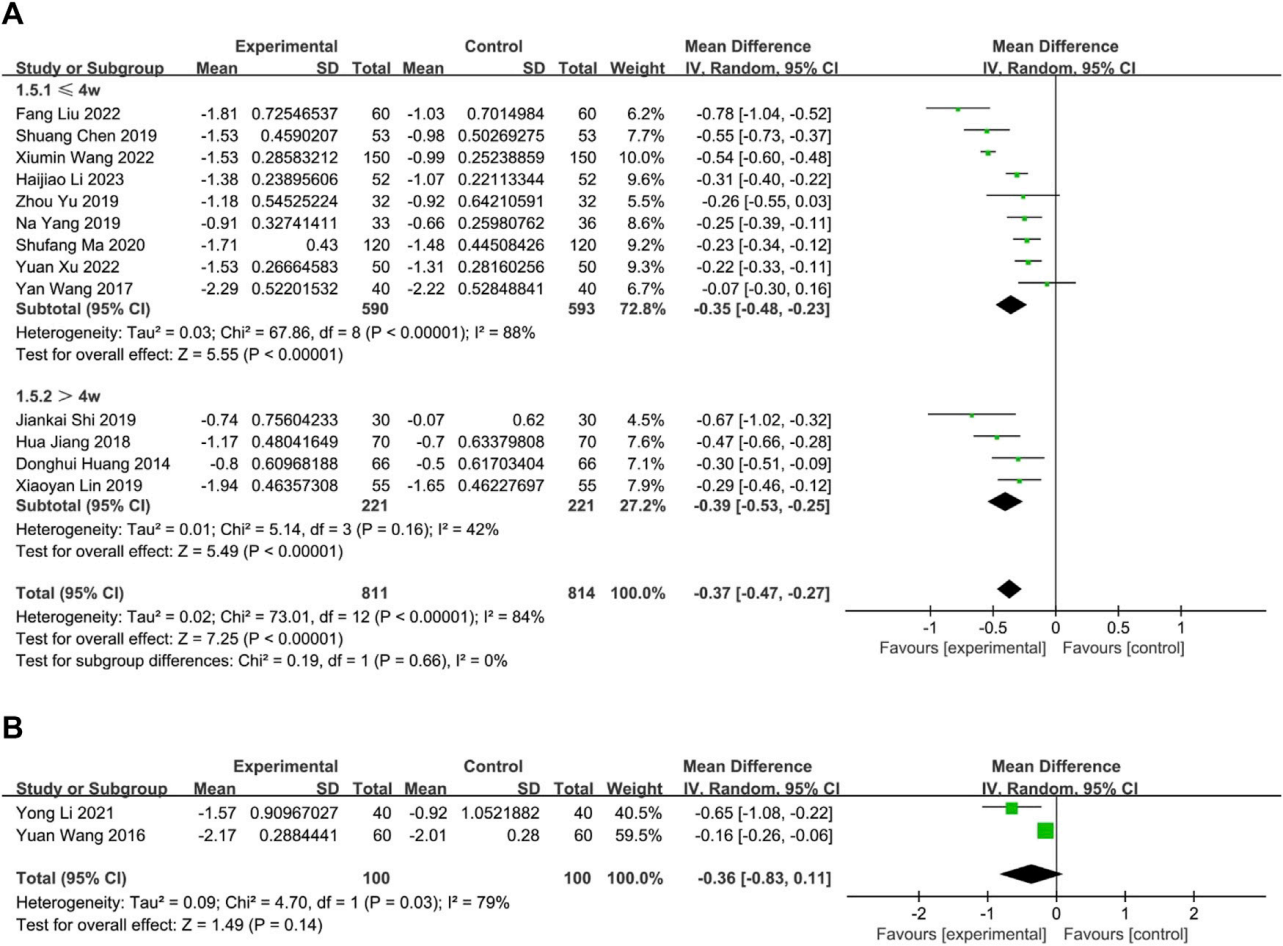
The result of meta-analysis of Combination of CHM and WM *versus* WM alone. **(A)** oral CHM; **(B)** external CHM.

Thirteen trials compared the effectiveness of combining oral CHM with WM to that of WM alone. Based on a random-effects model analysis (*p* < 0.00001, I2 = 84%), the CHM group demonstrated a statistically significant advantage over the WM group (*n* = 1,625, MD = −0.37, 95% CI: −0.47 to −0.27, *p* < 0.00001; Figure 6A). Of the thirteen RCTs, nine (Wang, 2017; Chen, 2019; Yang et al., 2019; Yu and Wang, 2019; Ma, 2020; Wang X. et al., 2022; Liu and Yang, 2022; Xu and Chen, 2022; Li et al., 2023) had interventions lasting no more than 4 weeks (*p* < 0.00001, I2 = 84%), while four (Huang et al., 2014; Jiang et al., 2018; Lin, 2019; Shi et al., 2019) had interventions lasting more than 4 weeks (*p* = 0.16, I2 = 42%). The subgroup analysis results were consistent with the overall findings (*n* = 1,183; MD = −0.35; 95% CI: −0.48 to −0.23; *p* < 0.00001; n = 442; MD = −0.39; 95% CI: −0.53 to −0.25; *p* < 0.00001; Figure 6A). No publication bias was found by Begg’s test (*p* = 0.30; Supplementary Figure S1). Sensitivity analysis was performed with one-by-one exclusion and the results of the meta-analysis were found to be stable (Supplementary Figure S2).

###### 3.3.1.3.2 External treatment with CHM

In two RCTs (Wang and Zhao, 2016; Li and Guo, 2021), external CHM combined with WM was compared to WM alone, with an intervention period lasting up to 4 weeks. Although the results of both RCTs indicated better efficacy of CHM in relieving nasal itching than WM, a random-effects model analysis did not show any significant difference between the two treatments (*p* = 0.03, I2 = 79%, *n* = 200, MD = −0.36, 95% CI: −0.83 to 0.11, *p* = 0.14; Figure 6B).

### 3.4 Secondary outcomes

#### 3.4.1 Effective rate

##### 3.4.1.1 Chinese herbal medicine *versus* placebo

**FIGURE 7:**
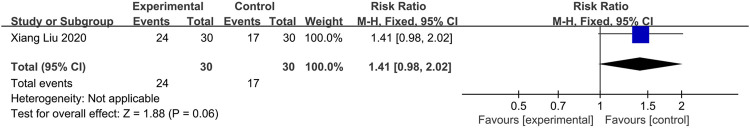
The result of meta-analysis of CHM *versus* placebo on effective rate.

One study (Liu et al., 2020) compared CHM with Placebo. Fixed-effects models showed no statistical difference between the two groups (*n* = 60, RR = 1.41, 95% CI: 0.98 to 2.02, *p* = 0.06; Figure 7).

##### 3.4.1.2 CHM *versus* WM

**FIGURE 8:**
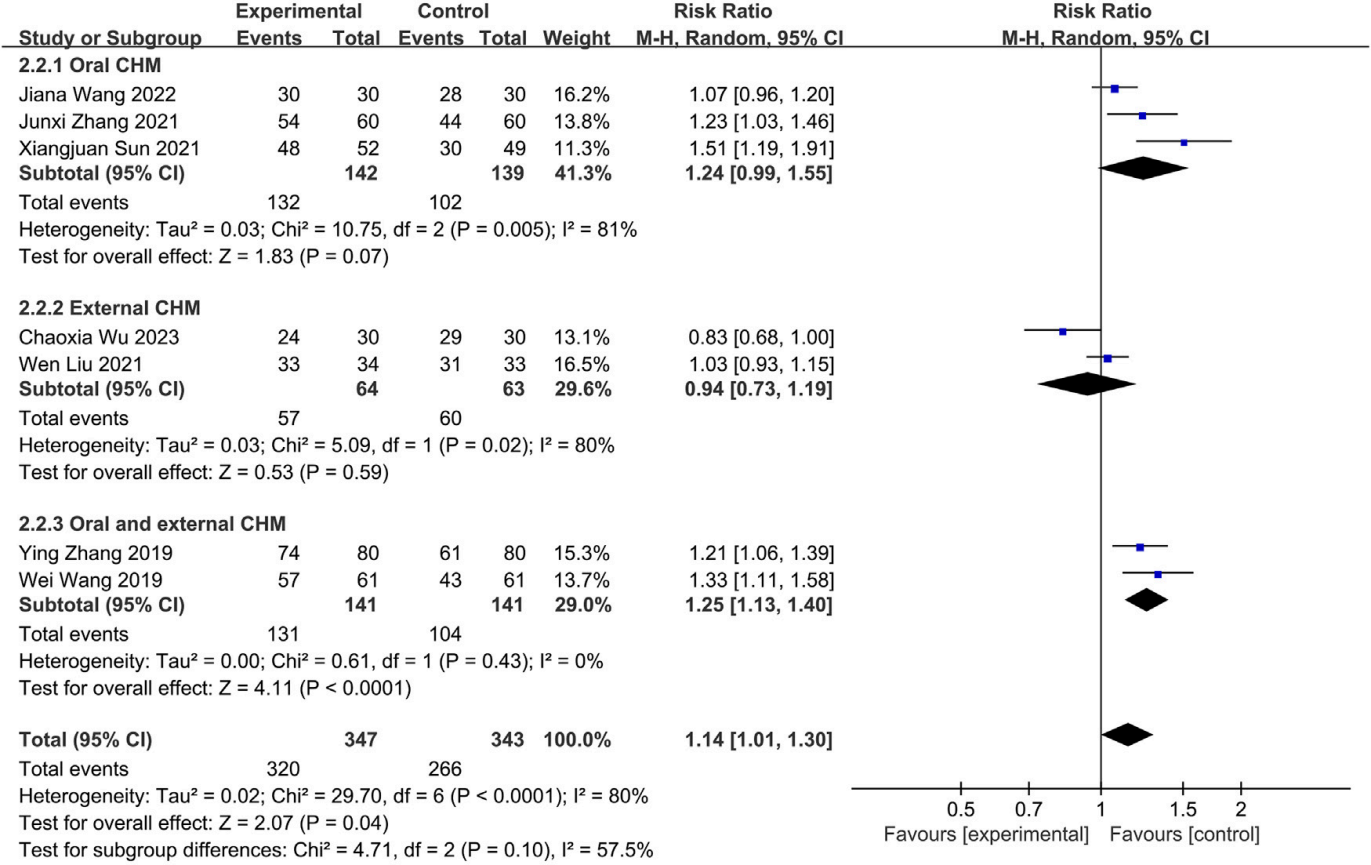
The result of meta-analysis of CHM *versus* WM on effective rate.

Seven studies compared CHM to WM. Of these, three (Sun et al., 2021; Zhang, 2021; Wang J. et al., 2022) investigated oral CHM, two (Liu W. et al., 2021; Wu et al., 2023) evaluated external CHM, and two (Wang et al., 2019; Zhang et al., 2019) assessed oral combined with external CHM. Based on a random-effects model analysis (*p* < 0.0001, I2 = 80%), there was no significant difference between oral CHM (n = 281, RR = 1.24, 95% CI: 0.99 to 1.55, *p* = 0.07; Figure 8) and external CHM (*n* = 127, RR = 0.94, 95% CI: 0.73 to 1.19, *p* = 0.59; Figure 8) and WM. Oral combined with external CHM (*n* = 282, RR = 1.25; 95% CI: 1.13 to 1.40; *p* < 0.0001; Figure 8) demonstrated a statistically significant advantage over WM.

##### 3.4.1.3 Combination of CHM and WM *versus* WM

**FIGURE 9:**
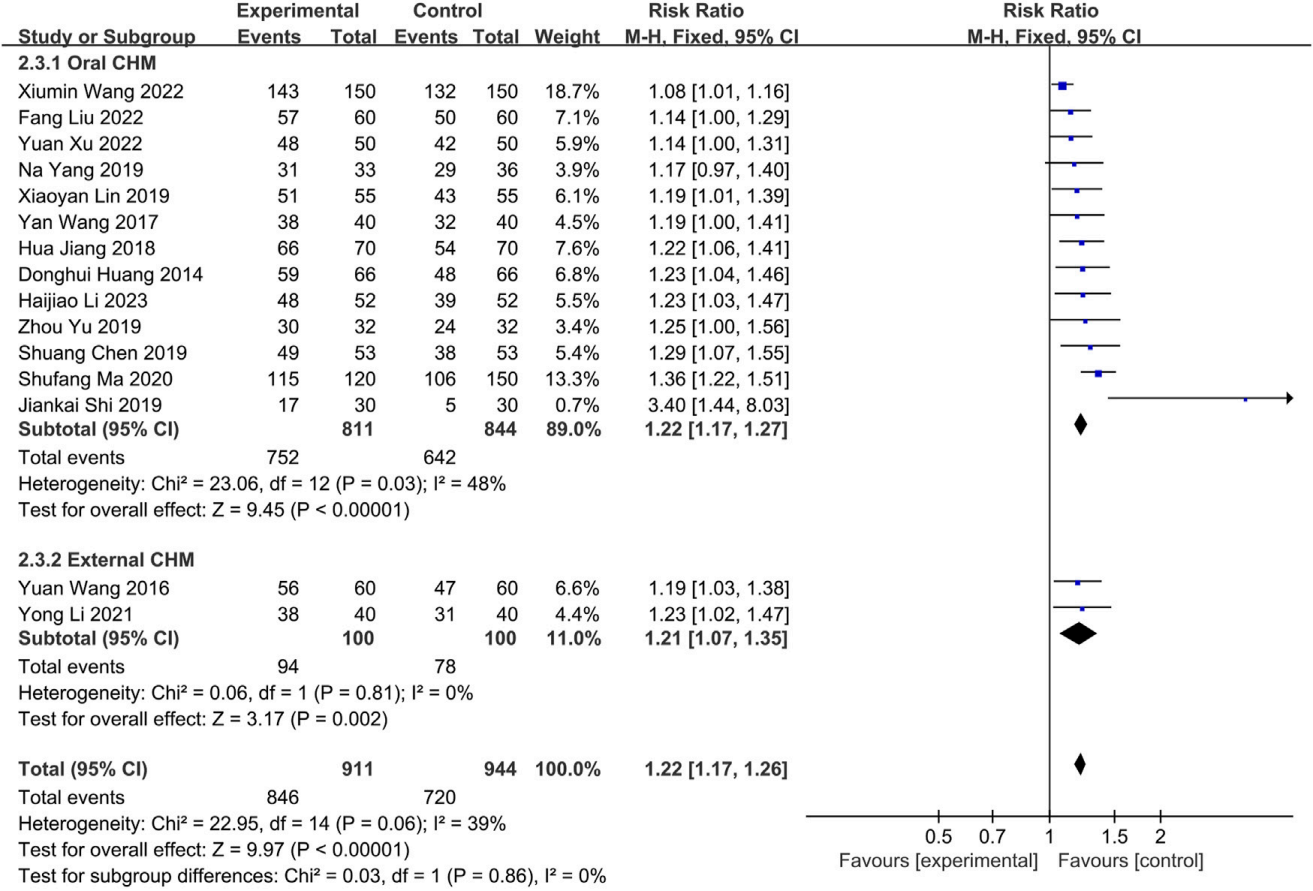
The result of meta-analysis of the combination of CHM and WM *versus* WM alone on effective rate.

Fifteen studies were conducted to compare the effectiveness of combining CHM with WM to WM alone. Thirteen (Huang et al., 2014; Wang, 2017; Jiang et al., 2018; Chen, 2019; Lin, 2019; Shi et al., 2019; Yang et al., 2019; Yu and Wang, 2019; Ma, 2020; Wang X. et al., 2022; Liu and Yang, 2022; Xu and Chen, 2022; Li et al., 2023) utilizing oral CHM and two (Wang and Zhao, 2016; Li and Guo, 2021) using external CHM were included in this comparison. A fixed-effects model (*p* = 0.11, I2 = 34%) demonstrated that both oral and external CHM combined with WM had a significant statistical advantage over WM alone (*n* = 1,625, RR = 1.18, 95% CI: 1.13 to 1.22, *p* < 0.00001; *n* = 200, RR = 1.21, 95% CI: 1.07 to 1.35, *p* = 0.002; Figure 9). Publication bias was found by Begg’s test (*p* = 0.006; Supplementary Figure S3).

#### 3.4.2 Serum IgE level

##### 3.4.2.1 CHM *versus* WM*

**FIGURE 10:**
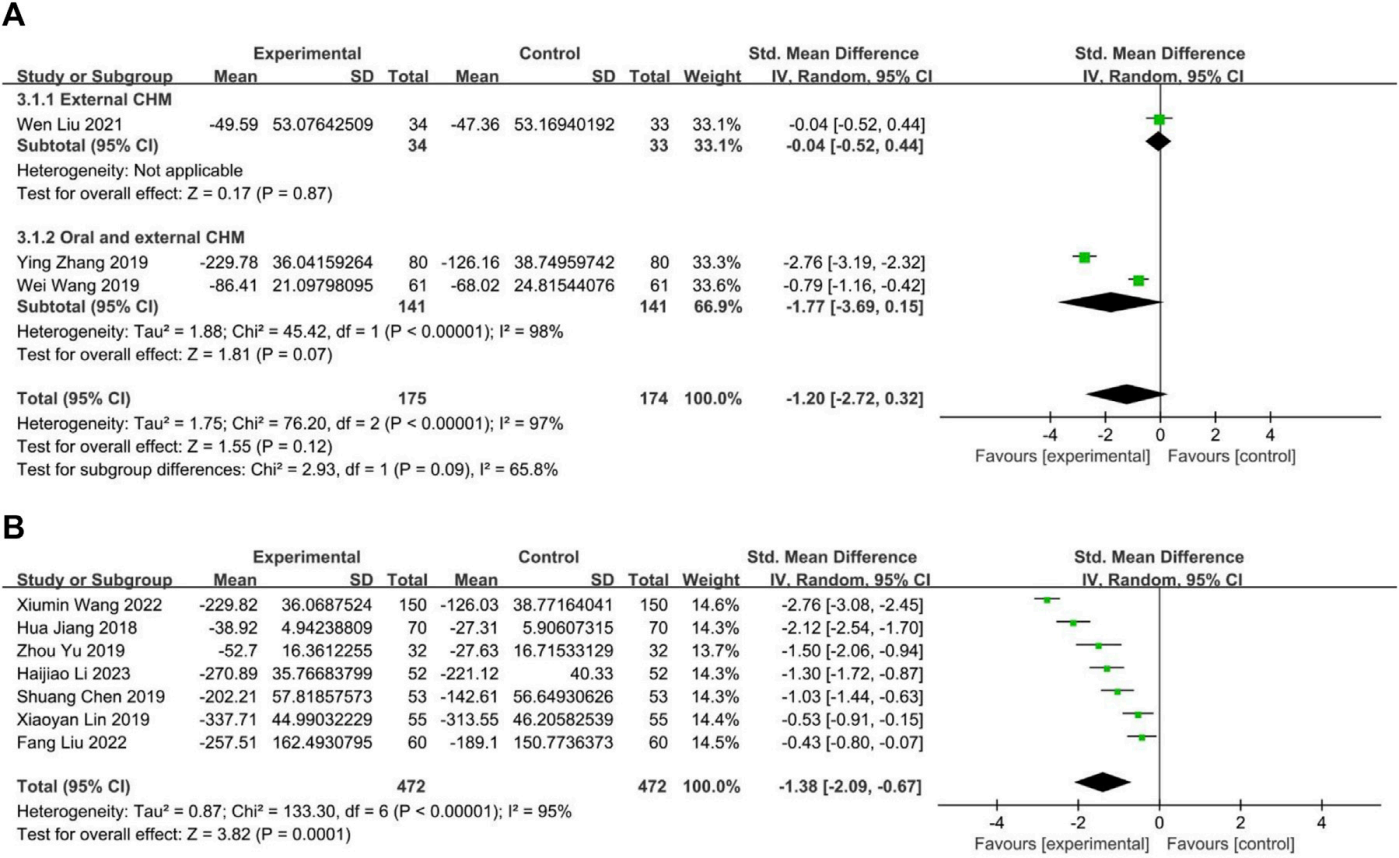
The result of meta-analysis of IgE. **(A)** CHM *versus* WM; **(B)** Combination of CHM and WM *versus* WM alone.

Three studies compared CHM to WM, with one (Liu W. et al., 2021) evaluating external CHM, while two (Wang et al., 2019; Zhang et al., 2019) assessing oral combined with external CHM. Based on a random-effects model analysis (*p* < 0.00001, I2 = 98%), there was insufficient evidence to suggest that external CHM was significantly different from WM in relieving nasal itching (*n* = 67, SMD = −0.04, 95% CI: −0.52 to 0.44, *p* = 0.87; Figure 10A). Oral combined with external CHM did not significantly differ from WM (*n* = 282, SMD = −1.77, 95% CI: −3.69 to 0.15, *p* = 0.07; Figure 10A).

##### 3.4.2.2 Combination of CHM and WM *versus* WM

Seven studies (Jiang et al., 2018; Chen, 2019; Lin, 2019; Yu and Wang, 2019; Wang X. et al., 2022; Liu and Yang, 2022; Li et al., 2023) compared combining oral CHM with WM to WM alone. The random-effects models (*p* < 0.00001, I2 = 95%) demonstrated a statistically significant advantage of CHM combined with WM over WM alone (*n* = 944, SMD = −1.38, 95% CI: −2.09 to −0.67, *p* = 0.0001; Figure 10B).

#### 3.4.3 Serum IL-4 level

**FIGURE 11:**
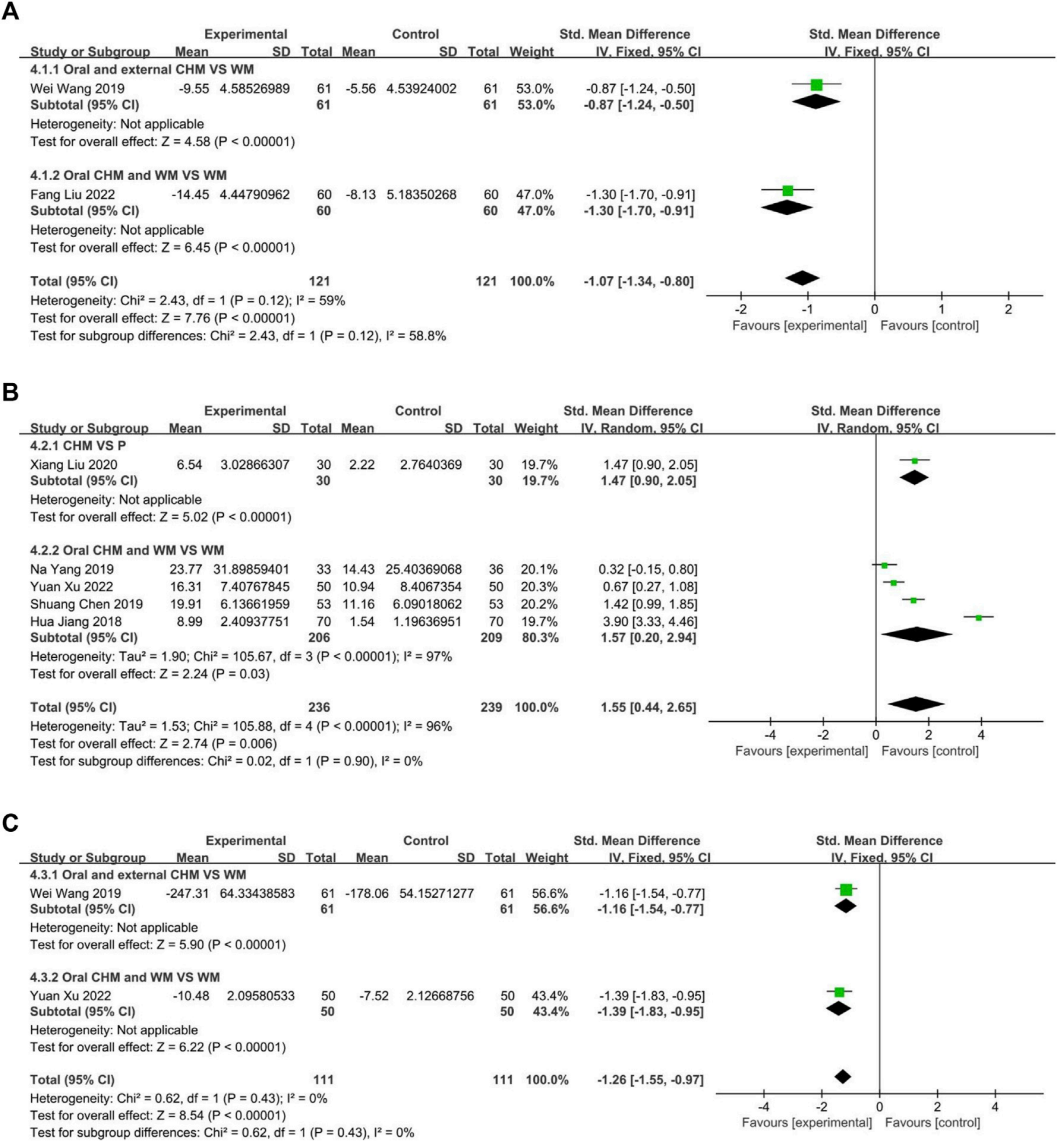
The result of meta-analysis of IL4, IL10, and IL33. **(A)** IL4; **(B)** IL10; **(C)** IL33.

Two studies measured the level of IL4, with one (Wang et al., 2019) comparing CHM to WM, and one (Liu and Yang, 2022) evaluated the efficacy of CHM combined with WM *versus* WM alone. The fixed-effect model (*p* = 0.12, I2 = 59%) demonstrated that both CHM alone and CHM combined with WM were significantly superior to WM in reducing IL4 levels (*n* = 122, SMD = −0.87, 95% CI: −1.24 to −0.50, *p* < 0.00001; n = 120, SMD = −1.30, 95% CI: −1.70 to −0.91, *p* < 0.00001; Figure 11A).

#### 3.4.4 Serum IL-10 level

Five trials reported IL10 levels, one (Liu et al., 2020) assessed CHM against Placebo, the remaining four studies (Jiang et al., 2018; Chen, 2019; Yang et al., 2019; Xu and Chen, 2022) evaluated the efficacy of combining CHM with WM *versus* WM alone. The result indicated that CHM is significantly related to decreased IL10 levels (*n* = 60, SMD = 1.47, 95% CI: 0.90 to 2.05, *p* < 0.00001; Figure 11B). A random-effect model (*p* < 0.00001, I2 = 96%) revealed a statistically significant advantage of combining CHM with WM over WM alone (*p* < 0.00001, I2 = 96%, n = 415, SMD = 1.57, 95% CI: 0.20 to 2.94, *p* < 0.00001; Figure 11B).

#### 3.4.5 Serum IL-33 level

This study analyzed two trials, one (Wang et al., 2019) comparing CHM to WM and the other (Xu and Chen, 2022) investigating the efficacy of combining CHM and WM *versus* WM alone. The fixed-effect model (*p* = 0.43, I2 = 0%) demonstrated that both CHM alone and in combination with WM resulted in significantly better outcomes than WM (*n* = 122, SMD = −1.16, 95% CI: −1.54 to −0.77, *p* < 0.00001; *n* = 100, SMD = −1.39, 95% CI: −1.83 to −0.95, *p* < 0.00001; Figure 11C).

#### 3.4.6 Recurrence rate

**FIGURE 12:**
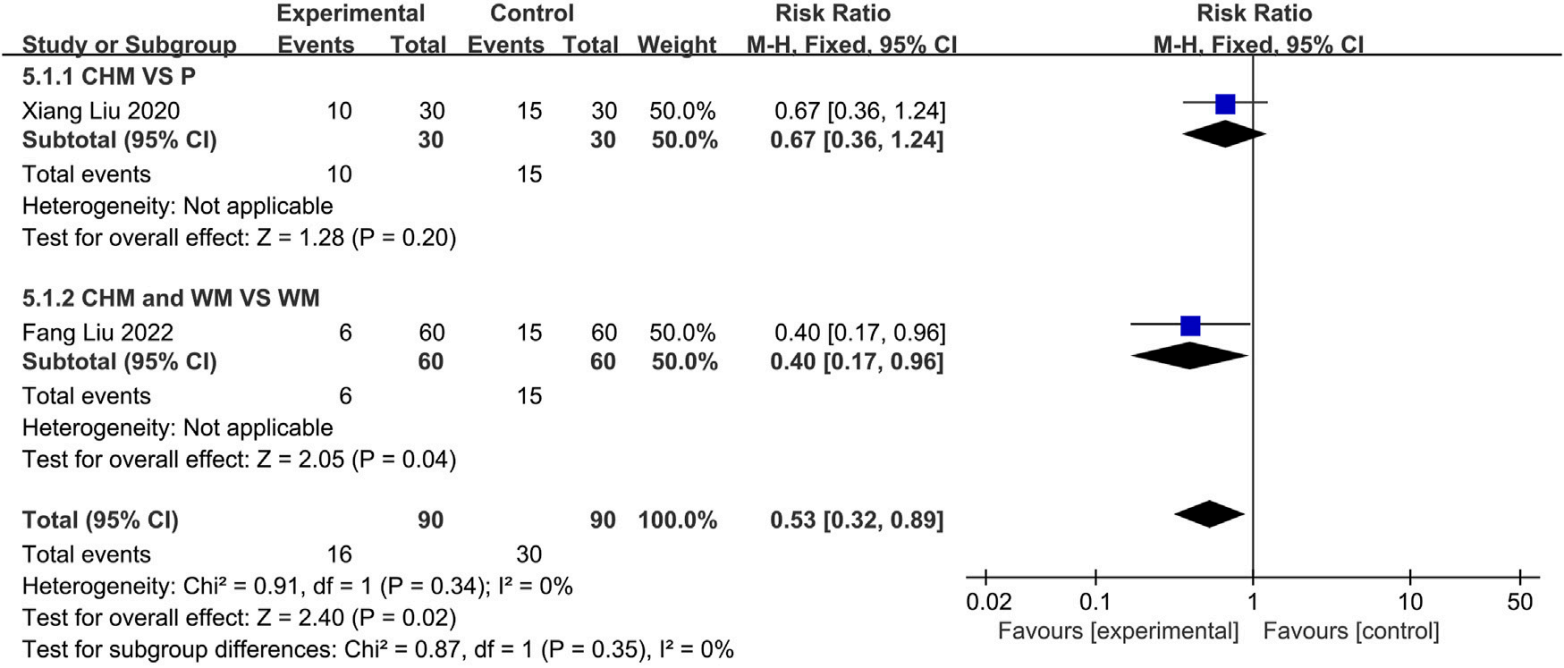
The result of meta-analysis of recurrent rate.

Two trials reported the recurrence rates. One(Liu et al., 2020) compared CHM to Placebo, and the other (Liu and Yang, 2022) compared the combination of CHM with WM to using WM alone. The fixed-effect (*p* = 0.34, I2 = 0%) models demonstrated no statistically significant difference between CHM and placebo groups (*n* = 60, RR = 0.67, 95% CI: 0.36 to 1.24, *p* = 0.20; Figure 12). However, a significant difference was observed between CHM with WM compared to WM alone, indicating lower recurrence rates among the former (*n* = 120, RR = 0.40, 95% CI: 0.17 to 0.96, *p* = 0.04; Figure 12).

#### 3.4.7 Safety

**FIGURE 13:**
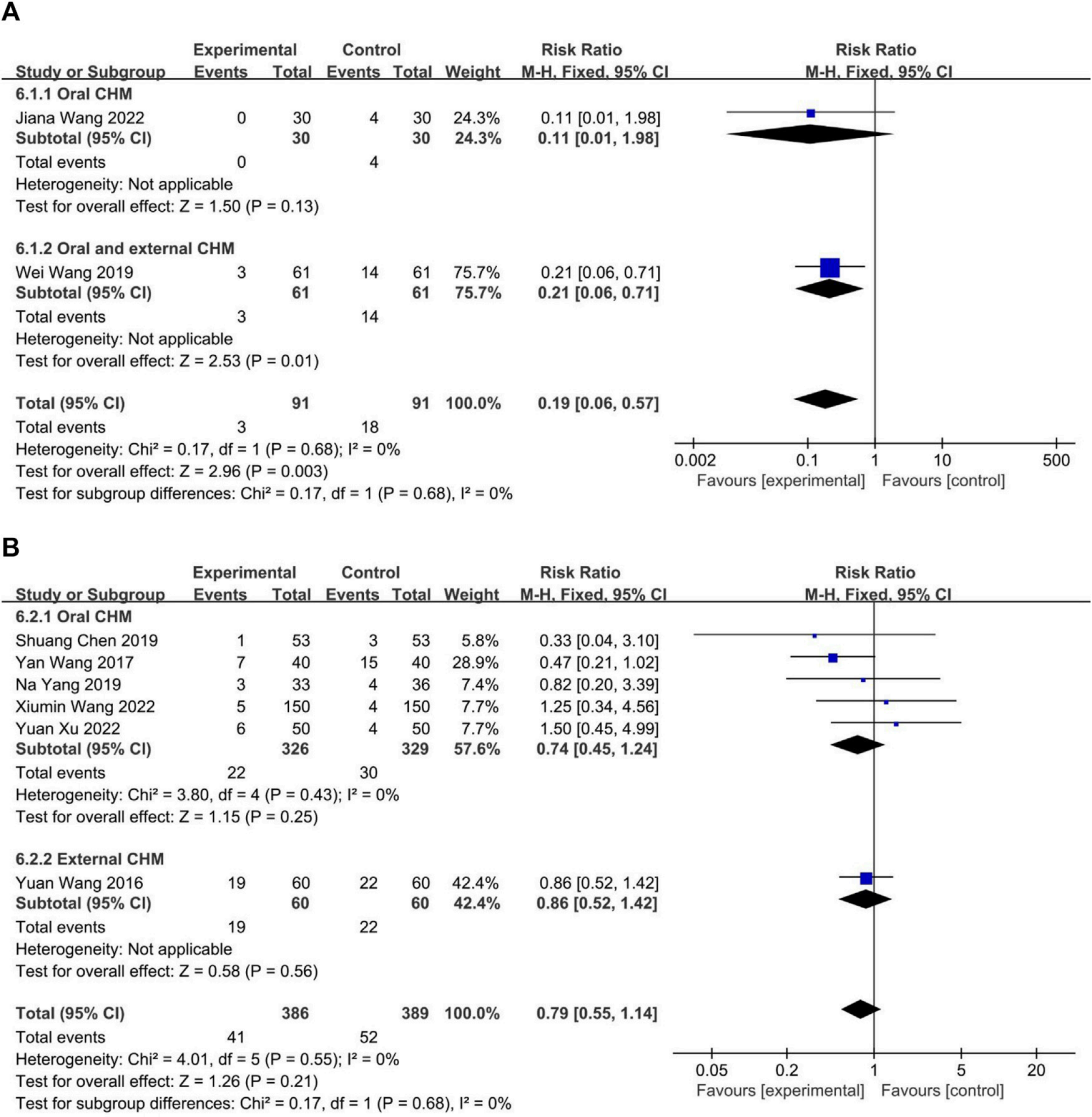
The result of meta-analysis of adverse reaction. **(A)** CHM *versus* WM; **(B)** Combination of CHM and WM *versus* WM alone.

The safety profiles of CHM and WM were evaluated in eight trials. Two (Wang et al., 2019; Wang J. et al., 2022) compared CHM to WM, and six (Wang and Zhao, 2016; Wang, 2017; Chen, 2019; Yang et al., 2019; Wang X. et al., 2022; Xu and Chen, 2022) compared combined CHM with WM to WM alone. The fixed-effect model (*p* = 0.68, I2 = 0%) indicated that CHM had a lower incidence of adverse drug reactions compared to WM (*p* = 0.68, I2 = 0%; *n* = 182, RR = 0.19, 95% CI: 0.06 to 0.57, *p* = 0.003; Figure 13A) and no significant difference between the combination of CHM with WM and WM alone (*p* = 0.55, I2 = 0%; *n* = 775, RR = 0.79, 95% CI: 0.55 to 1.14, *p* = 0.21; Figure 13B), according to the fixed-effect model (*p* = 0.68, I2 = 0%).

### 3.5 GRADE for the main comparisons

The GRADE quality of evidence for all outcomes was evaluated. The quality of Nasal itch, effective rate, IgE, IL10, IL33, and recurrent rate were low. The quality of IL4 was very low. The quality of adverse reaction was moderate (Supplementary Table S2).

### 3.6 Description of CHM

A total of 68 different herbal medicines were used in 23 RCTs. Eight herbals were used more than seven times (10%), includes Biond Magnolia Immature Flower, Liquorice Root, Membranous Milkvetch Root, Divaricate Saposhnikovia Root, Siberian Cocklebur Fruit, Dahurian Angelica Root, Peppermint Rhizome, and Largehead Atractylodes Rhizome (Supplementary Table S3).

## 4 Discussion

### 4.1 Summary of evidence

Chinese herbal medicine is a widely used treatment for allergic rhinitis in children in China. Although previous Chinese studies have demonstrated the therapeutic effects of CHM on allergic rhinitis in children, their level of evidence was low. Therefore, we conducted a meta-analysis of 23 randomized controlled trials (RCTs) with 2,605 children to pool the information on CHM’s efficacy for nasal itching symptom relief, modulation of immune imbalance (IgE, IL-4, IL-10, and IL-33), relapse reduction, and safety.

The risk of bias assessment indicates the need for attention to the risk of bias in the randomization process and selective reporting of endpoints in the included studies. This concern primarily arises from the absence of blinding, lack of reporting on specific methods of random allocation concealment, and failure to report study protocols.

Our primary findings indicate that Chinese herbal medicine (CHM) is related to reducing nasal itching and raising interleukin-10 (IL-10) levels compared to placebo. When administered orally, herbal medicines alone alleviate nasal itching and suppress levels of immunoglobulin E (IgE), interleukin-4 (IL-4), and interleukin-33 (IL-33), while simultaneously increasing IL-10 levels in contrast to Western medicines. Additionally, the combination of oral and topical CHM substantially alleviates nasal itching. External CHM was overall similar to WM in relieving nasal itching, efficiency, and lowering IgE levels. The combined external application of CHM and WM did not differ statistically from using WM alone for relieving nasal itching. Both studies within the subgroup indicated that external CHM combined with WM was superior in relieving nasal itching, but the meta-analysis indicated the opposite result. This was due to the use of a randomized effect model to account for the high heterogeneity. When using a fixed-effects model can obtain a more significant result. Therefore, we believe that topical CHM combined with WM is beneficial in the treatment of nasal itching. Statistically significant advantages were also observed when CHM was used with WM in other indicators. For the same reason, we believe that oral combined with external CHM is effective in reducing IgE. Eight randomized controlled trials (RCTs) reported adverse effects such as headache, dizziness, malaise, and dry mouth; however, all self-heal and no serious adverse events were reported. Meta-analysis results suggest that CHM alone or combined with WM has a favorable safety profile.

We found that the results of using external CHM alone varied widely across studies. The effectiveness of external CHM may depend on the duration of intervention and the use of different WM controls. External CHM was more effective when the duration of treatment was longer than 4 weeks, with the control group using antihistamines. Conversely, when the duration of treatment was shorter, and the control group received glucocorticoids, the opposite result was observed. Nasal hormones act on glucocorticoid receptors in the nasal mucosa by reducing inflammatory factors and inhibiting inflammation cells, thus decreasing inflammatory factor-mediated hyperirritability of the nasal mucosa. These two explanations may account for the lack of efficacy of external CHM.

### 4.2 Implications for practice

We have compiled comprehensive information regarding the herbal medicines utilized in this study, encompassing their types, frequency of use, and the outcomes associated with CHM prescriptions incorporated within commonly used herbal medicines. The presented results are detailed below: Across the 23 studies analyzed, the detailed composition of the CHM varied widely and included over 60 different herbs. The most frequently used CHMs were *Magnolia denudate* Desr, *Glycyrrhiza glabra* L, *Astragalus mongholicus* Bunge, *Saposhnikovia divaricate* (Turcz. ex Ledeb.) Schischk, *Xanthium strumarium* L, *Angelica dahurica* (Hoffm.) Benth. & Hook. f. ex Franch. & Sav, *Mentha canadensis* L, *Atractylodes macrocephala* Koidz. Cang Erzi San (*Xanthium strumarium* L, *Magnolia denudate* Desr, *Angelica dahurica* (Hoffm) Benth. & Hook. f. ex Franch. & Sav, *Mentha canadensis* L) and Yu Pingfeng San (*Astragalus mongholicus* Bunge, *Atractylodes macrocephala* Koidz., *Saposhnikovia divaricate* (Turcz. ex Ledeb.) Schischk) are most commonly used CHM redescription for treating allergic rhinitis. The present findings may have important implications for the TCM management of rhinitis in children, informing the development of relevant guidelines.

### 4.3 Limitations of the study

This study conducted a thorough and comprehensive literature search to examine the efficacy and safety of Chinese herbal medicine (CHM) therapy for children with allergic rhinitis. The findings indicate that CHM holds significant potential in alleviating symptoms of nasal itchiness, modulating inflammatory responses, and reducing recurrence.

However, the study still has several limitations. Firstly, the methodological quality of the included studies was low. Only one study implemented blinding, and no study reported trial protocol, making determining adherence to the prescribed protocols challenging. Most studies did not explicitly report the concealment of random assignment. Secondly, the assessment of nasal itchiness relied on subjective measures, while the blood test results were objective. However, different studies used varying units for measurement, making it challenging to fully mitigate heterogeneity, despite using Delta values and standardized mean differences (SMDs). Thirdly, substantial variability in the composition of Chinese herbal medicine (CHM) prescriptions and administration methods contributed to heterogeneity across the studies. Fourthly, most of the included studies did not evaluate the long-term efficacy of CHM. Given the association between allergic rhinitis and other conditions such as asthma and ADHD, it is essential to consider the co-occurrence of these diseases when assessing the long-term efficacy of CHM. Lastly, it is essential to note that all the included studies were conducted in single-centre settings in China, potentially introducing publication bias.

### 4.4 Implications for research

Based on the findings and limitations, we propose the following key considerations for future research: Firstly, it is crucial to enhance the methodological quality of studies. This can be achieved by pre-registering the study protocol, ensuring data transparency, and rigorously implementing randomization, allocation concealment, and blinding throughout the study process to uphold the integrity of the research. Secondly, it is crucial to improve the design of clinical studies by selecting more objective outcome measures, authenticating the data, and minimizing individual differences. Additionally, incorporating appropriate follow-up periods that align with the characteristics of the disease, such as monitoring recurrence rates and associated comorbidities in the case of rhinitis, will provide valuable clinical insights. Thirdly, for randomized controlled trials involving Chinese herbal medicine (CHM), adherence to the CONSORT Extension for Chinese Herbal Medicine Formulas 2017 (Cheng et al., 2017) is recommended to ensure standardization and authenticity of the trials. Fourthly, implementing multicenter, large-sample, and high-quality clinical studies is warranted to enhance the generalizability of research findings. Fifth, in view of the research potential of Chinese herbal medicines in allergic diseases, clinical and animal experimental studies focusing on the active ingredients of Chinese herbal medicines may elucidate the specific effects and intrinsic mechanisms of different Chinese herbal medicinal ingredients and Chinese herbal prescriptions, to better guide the use of CHM in pediatric rhinitis. For example, studies (Liu et al., 2023) have confirmed that the CHM prescription YPF and its main active compound wogonin may alleviate airway inflammation in asthma by inhibiting PI3K/AKT, IL-17 and TNF-α signaling pathways.

Given the distinctive prescriptions and dosage forms of CHM, achieving blinding in clinical research involving CHM presents a challenging task. This study identified two potential common CHM prescriptions and eight herbal medicines for childhood rhinitis. It is worth considering future investigations to explore the feasibility of utilizing these prescriptions and drugs to develop new CHM dosage forms. This would facilitate blinding in clinical studies of childhood rhinitis and enhance the completeness of research protocols for RCTs. This study included 12 RCTs, which reported similar TCM evidence. Subgroup analysis of TCM evidence demonstrated an association between CHM use and a reduction in itch (Supplementary Figure S4). However, due to the overall low methodological quality of the original studies included in this systematic review, we believe that additional high-quality basic and clinical studies are necessary to further validate the role of herbal and CHM prescriptions, as well as traditional Chinese medicine (TCM) evidence, in clinical practice.

## 5 Conclusion

Chinese herbal medicine (CHM) holds great potential in alleviating symptoms, modulating immune factors levels, and reducing relapse in pediatric rhinitis. Meanwhile, CHM is relatively safe. However, the efficacy and safety of CHM in treating pediatric rhinitis still need to be confirmed due to the inclusion of studies with low methodological quality, small sample sizes, and potential heterogeneity. More large-sample, high-quality RCTs are necessary to provide reliable evidence for the clinical application of CHM.

## Data Availability

The original contributions presented in the study are included in the article/Supplementary Material, further inquiries can be directed to the corresponding author.
