# Supplementary figures and images for: Efficacy of Chinese herbal medicine on nasal itching in children with allergic rhinitis: a systematic review and meta-analysis
Source: Front Pharmacol. 2023 Aug 23;14:1240917. doi: 10.3389/fphar.2023.1240917 (PMC10482051; doi:10.3389/fphar.2023.1240917)

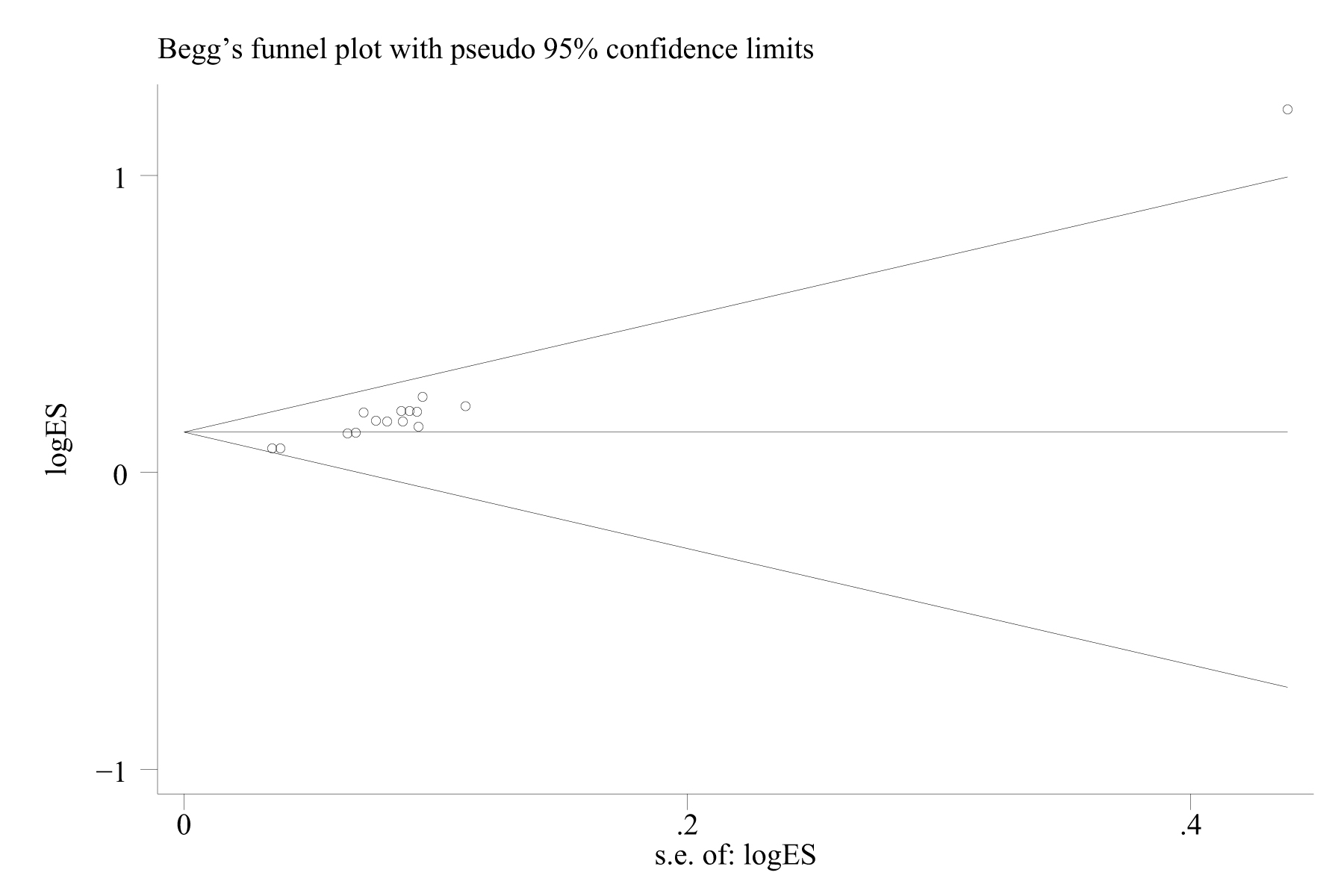

Supplement: Supplementary file 1 [file Image3.JPEG]

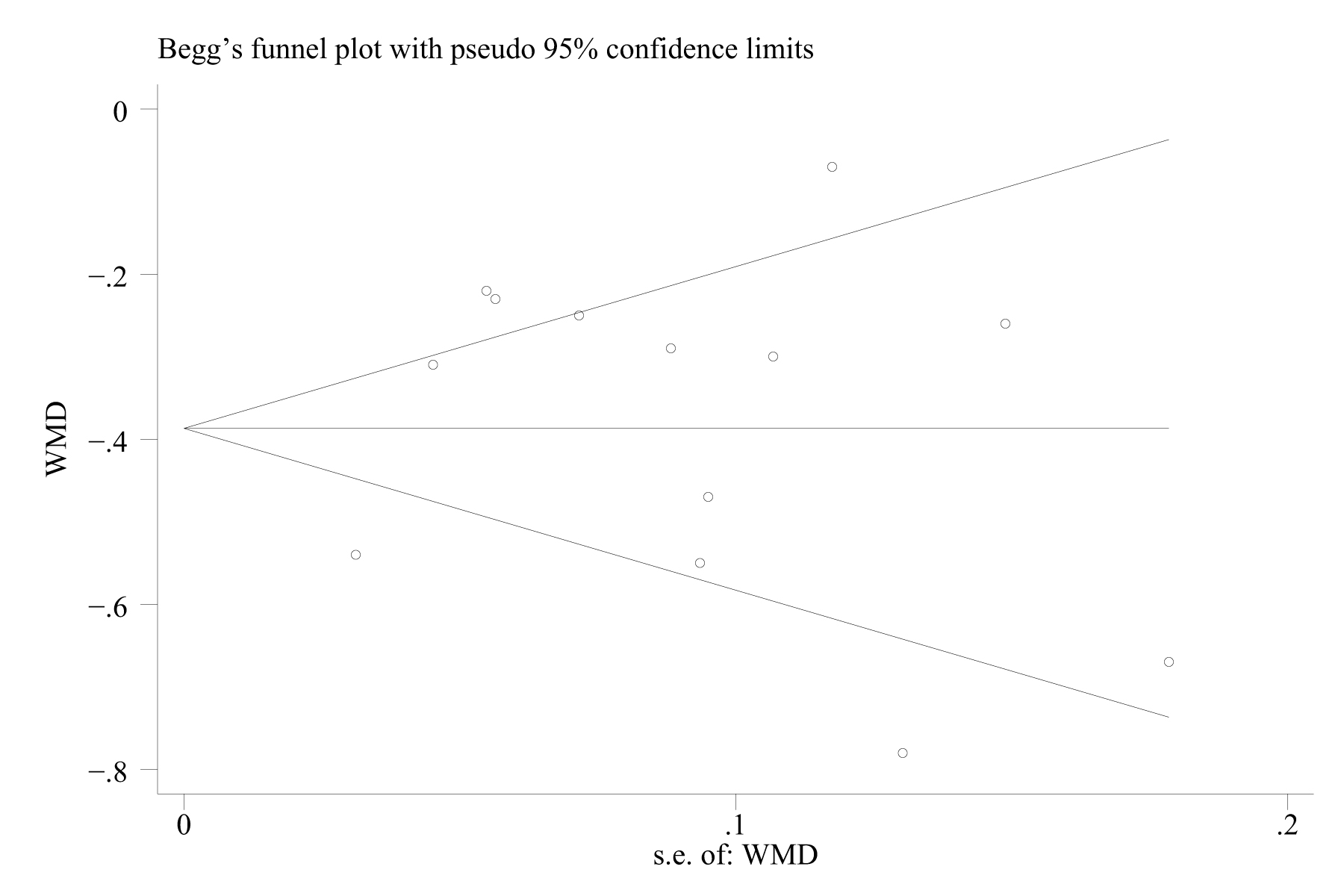

Supplement: Supplementary file 3 [file Image1.JPEG]

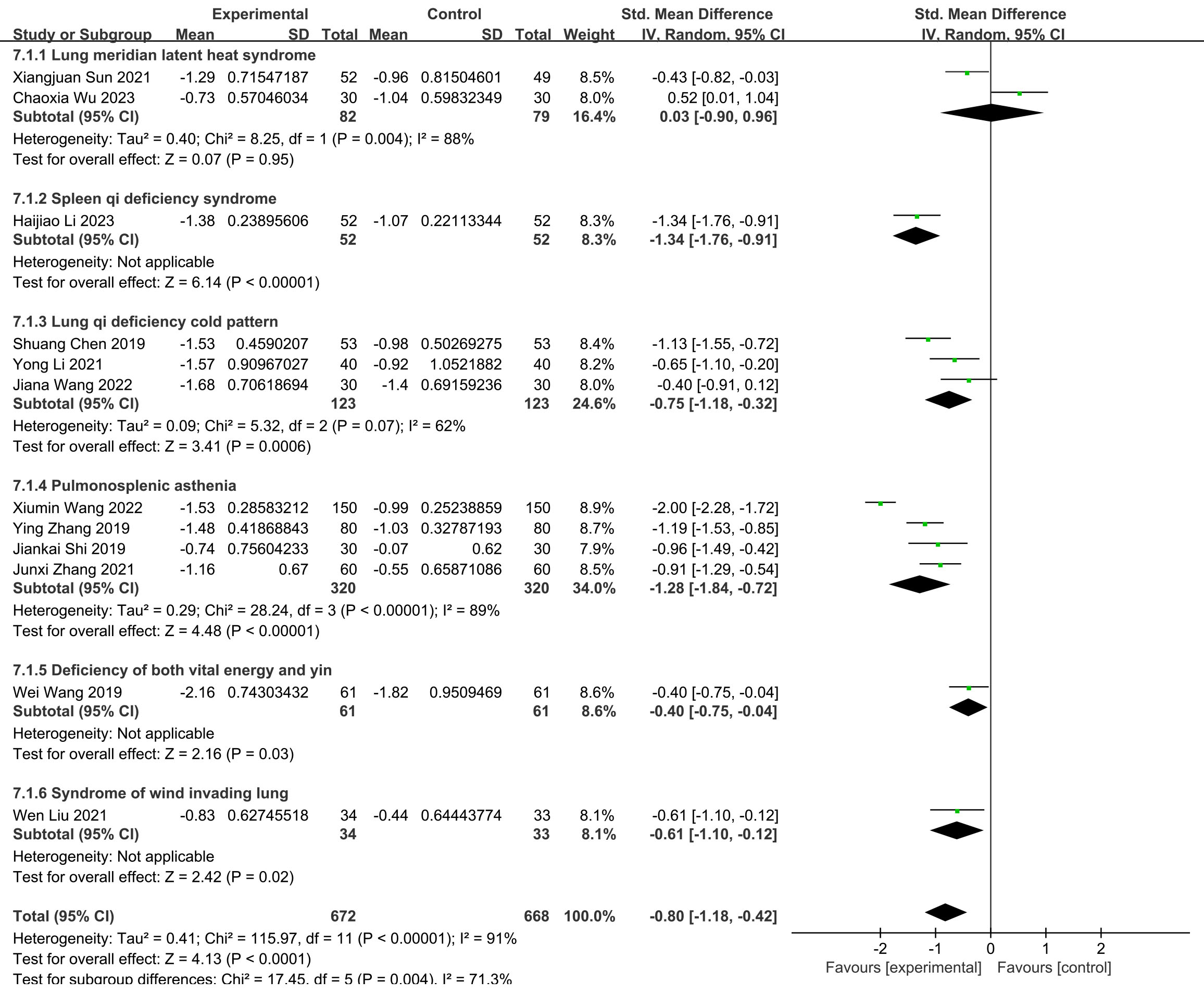

Supplement: Supplementary file 4 [file Image4.JPEG]

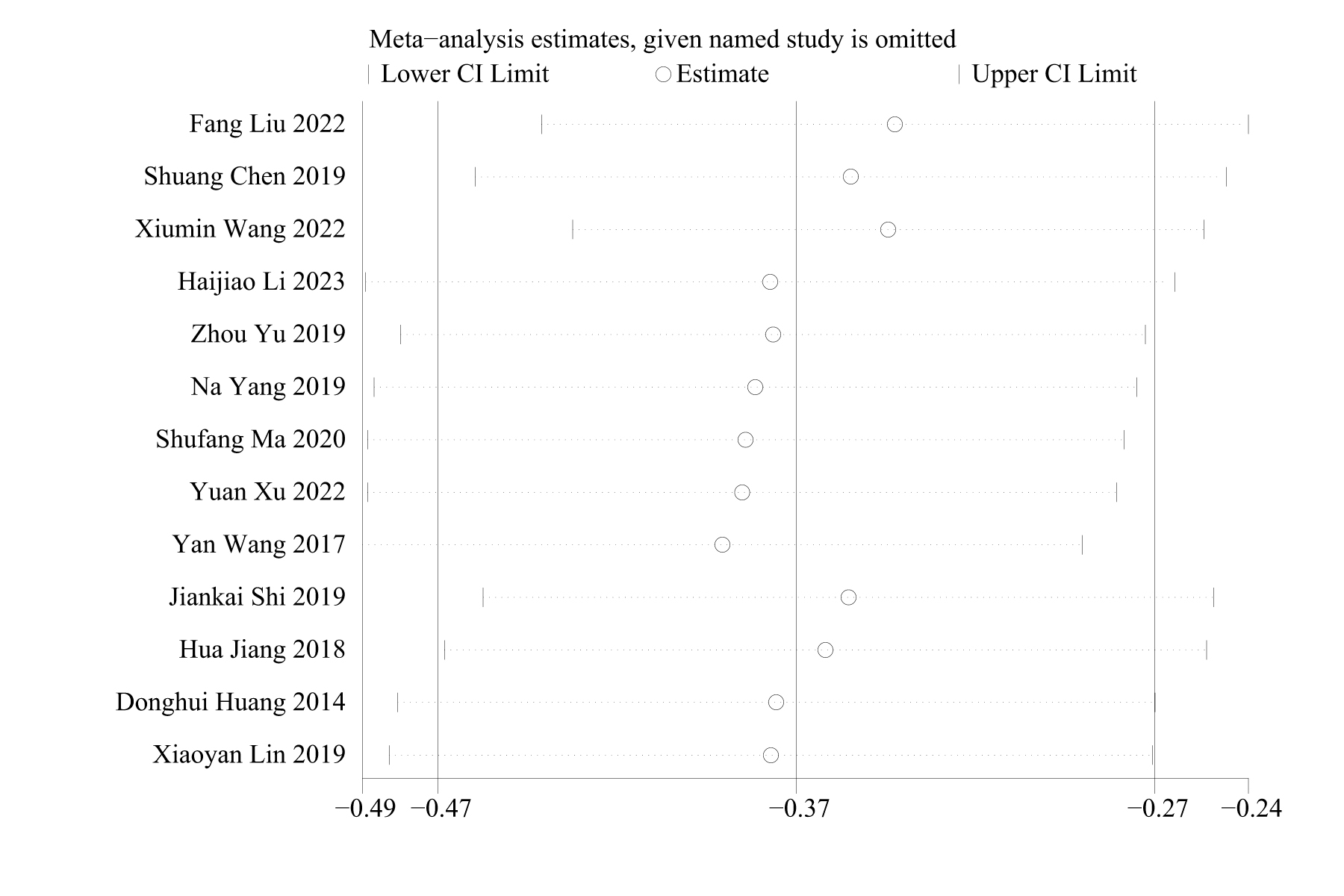

Supplement: Supplementary file 5 [file Image2.JPEG]
