# Supplementary material for: Efficacy of Chinese herbal medicine on nasal itching in children with allergic rhinitis: a systematic review and meta-analysis
Source: Front Pharmacol. 2023 Aug 23;14:1240917. doi: 10.3389/fphar.2023.1240917 (PMC10482051; doi:10.3389/fphar.2023.1240917)
Supplement: Supplementary file 2 [file Table1.DOCX]

**Supplementary Table 1 Supplementary information of all studies**

| **Study ID** | **Source of patients** | **TCM syndromes** | **Sources of funding** |
| --- | --- | --- | --- |
| Chaoxia Wu 2023 | Outpatient department | Lung meridian latent heat syndrome | Fund Project of Affiliated Hospital of Chengdu University of Traditional Chinese Medicine ( No.20ZJ05 ) |
| Donghui Huang 2014 | Outpatient department | NA | NA |
| Fang Liu 2022 | NA | NA | NA |
| Haijiao Li 2023 | NA | Spleen qi deficiency syndrome | Taizhou Science and Technology Bureau 2020 Taizhou second batch of social development science and technology plan project ( 20ywb93 ) |
| Hua Jiang 2018 | NA | NA | NA |
| Jiana Wang 2022 | Outpatient department | Lung-qi deficiency cold pattern | NA |
| Jiankai Shi 2019 | Outpatient department | Pulmonosplenic asthenia | NA |
| Junxi Zhang 2021 | NA | Pulmonosplenic asthenia | NA |
| Na Yang 2019 | NA | NA | NA |
| Shuang Chen 2019 | NA | Lung-qi deficiency cold pattern | NA |
| Shufang Ma 2020 | Outpatient department | NA | NA |
| Wei Wang 2019 | Outpatient department | Deficiency of both vital energy and yin | Hebei Provincial Administration of Traditional Chinese Medicine 2019 Chinese Medicine Research Plan Project ( No.2019236 ) |
| Wen Liu 2021 | Outpatient department | Syndrome of wind invading lung | Scientific research project of Fujian Provincial Department of Education ( JAT170279 ) ; Fujian Provincial Health and Family Planning Commission Youth Scientific Research Project ( 2017-1-75 ) |
| Xiang Liu 2020 | NA | NA | Scientific research project of Foshan Municipal Health and Family Planning Bureau of Guangdong Province ( No.20180106 ) ; Foshan Science and Technology Bureau Research Project ( No.1920001001284, 2016AB002121 ) |
| Xiangjuan Sun 2021 | Outpatient department | Lung meridian latent heat syndrome | Science and Technology Development Fund of Affiliated Hospital of Chengdu University of Traditional Chinese Medicine ( 2011-D-YY-24, Y2019154 ) |
| Xiaoyan Lin 2019 | NA | NA | NA |
| Xiumin Wang 2022 | NA | Pulmonosplenic asthenia | Medical Research Project of Jiangsu Provincial Health Commission ( No. H2019137 ) ; Kunshan Science and Technology Plan Project ( No. KS18031 ) |
| Yan Wang 2017 | NA | NA | NA |
| Ying Zhang 2019 | NA | Pulmonosplenic asthenia | Hebei Province Scientific and Technological Achievements Evaluation Project : No.153 ( 2018 ) |
| Yong Li 2021 | NA | Lung-qi deficiency cold pattern | NA |
| Yuan Wang 2016 | Department of outpatient and inpatient department | NA | Natural Science Foundation of Liaoning Province ( 201102290 ) |
| Yuan Xu 2022 | Outpatient department | NA | NA |
| Zhou Yu 2019 | NA | NA | NA |
