# Supplementary material for: Efficacy of Chinese herbal medicine on nasal itching in children with allergic rhinitis: a systematic review and meta-analysis
Source: Front Pharmacol. 2023 Aug 23;14:1240917. doi: 10.3389/fphar.2023.1240917 (PMC10482051; doi:10.3389/fphar.2023.1240917)
Supplement: Supplementary file 6 [file Table2.DOCX]

**Supplementary Table 2 Summary of GRADE.**

| **Quality assessment** | | | | | | | **No of patients** | | **Effect** | | **Quality** | **Importance** |
| --- | --- | --- | --- | --- | --- | --- | --- | --- | --- | --- | --- | --- |
| **No of studies** | **Design** | **Risk of bias** | **Inconsistency** | **Indirectness** | **Imprecision** | **Other considerations** | **Nasal itching** | **Control** | **Relative (95% CI)** | **Absolute** |  |  |
| nasal itching (23 studies) | randomised trials | serious1 | serious2 | no serious indirectness | no serious imprecision | none | 1288 | 1287 | - | MD 0.36 lower (0.44 to 0.27 lower) | ÅÅOO | IMPORTANT |
|  |  |  |  |  |  |  |  |  |  |  | LOW |  |
| effective rate (23 studies) | randomised trials | serious1 | serious2 | no serious indirectness | no serious imprecision | none | 1190/1288 | 1003/1317 | RR 1.19 (1.13 to 1.25) | 145 more per 1000 (from 99 more to 190 more) | ÅÅOO | IMPORTANT |
|  |  |  |  |  |  |  | -92.40% | -76.20% |  |  | LOW |  |
|  |  |  |  |  |  |  |  | 77.10% |  | 146 more per 1000 (from 100 more to 193 more) |  |  |
| IgE (10 studies) | randomised trials | serious1 | serious2 | no serious indirectness | no serious imprecision | reporting bias4 | 647 | 646 | - | SMD 1.33 lower (1.95 to 0.71 lower) | ÅÅOO | IMPORTANT |
|  |  |  |  |  |  |  |  |  |  |  | LOW |  |
| IL4 (2 studies) | randomised trials | serious1 | serious2 | no serious indirectness | serious3 | none | 121 | 121 | - | SMD 1.08 lower (1.5 to 0.66 lower) | ÅOOO | IMPORTANT |
|  |  |  |  |  |  |  |  |  |  |  | VERY LOW |  |
| IL10 (5 studies) | randomised trials | serious1 | serious2 | no serious indirectness | no serious imprecision | none | 236 | 239 | - | SMD 1.55 higher (0.44 to 2.65 higher) | ÅÅOO | IMPORTANT |
|  |  |  |  |  |  |  |  |  |  |  | LOW |  |
| IL33 (2 studies) | randomised trials | serious1 | no serious inconsistency | no serious indirectness | serious3 | none | 111 | 111 | - | SMD 1.26 lower (1.55 to 0.97 lower) | ÅÅOO | IMPORTANT |
|  |  |  |  |  |  |  |  |  |  |  | LOW |  |
| recurrent rate (2 studies) | randomised trials | serious1 | no serious inconsistency | no serious indirectness | serious3 | none | 16/90 | 30/90 | RR 0.53 (0.32 to 0.89) | 157 fewer per 1000 (from 37 fewer to 227 fewer) | ÅÅOO | IMPORTANT |
|  |  |  |  |  |  |  | -17.80% | -33.30% |  |  | LOW |  |
|  |  |  |  |  |  |  |  | 37.50% |  | 176 fewer per 1000 (from 41 fewer to 255 fewer) |  |  |
| adverse reaction (8 studies) | randomised trials | serious1 | no serious inconsistency | no serious indirectness | no serious imprecision | none | 44/477 | 70/480 | RR 0.63 (0.45 to 0.89) | 54 fewer per 1000 (from 16 fewer to 80 fewer) | ÅÅÅO | IMPORTANT |
|  |  |  |  |  |  |  | -9.20% | -14.60% |  |  | MODERATE |  |
|  |  |  |  |  |  |  |  | 12.20% |  | 45 fewer per 1000 (from 13 fewer to 67 fewer) |  |  |
| 1 Lacking blinding and randomization and allocation are unclear; 2 Substantial heterogeneity; 3 Small sample size; 4 Publication bias | | | | | | | | | | | | |
