# Supplementary material for: Efficacy of Chinese herbal medicine on nasal itching in children with allergic rhinitis: a systematic review and meta-analysis
Source: Front Pharmacol. 2023 Aug 23;14:1240917. doi: 10.3389/fphar.2023.1240917 (PMC10482051; doi:10.3389/fphar.2023.1240917)
Supplement: Supplementary file 7 [file Table3.DOCX]

**Supplementary Table 3 The eight common used Chinese herbs of included studies.**

| Chinese name | Number of Studies(%) | Accept name | English name | Family |
| --- | --- | --- | --- | --- |
| xinyi | 16(24) | Magnolia denudata Desr. | Biond Magnolia Immature Flower | Magnoliaceae |
| gancao | 13(19) | *Glycyrrhiza glabra L.* | Liquorice Root | Fabaceae |
| huangqi | 12(18) | Astragalus mongholicus Bunge | Membranous Milkvetch Root | Fabaceae |
| fangfeng | 11(16) | Saposhnikovia divaricata (Turcz. ex Ledeb.) Schischk. | Divaricate Saposhnikovia Root | Apiaceae |
| cangerzi | 10(15) | Xanthium strumarium L. | Siberian Cocklebur Fruit | Asteraceae |
| baizhi | 9(13) | Angelica dahurica (Hoffm.) Benth. & Hook.f. ex Franch. & Sav. | Dahurian Angelica Root | Apiaceae |
| bohe | 8(12) | Mentha canadensis L. | Reppermint Rhizome | Lamiaceae |
| baizhu | 7(10) | Atractylodes macrocephala Koidz. | Largehead Atractylodes Rhizome | Asteraceae |
